# Improvement of the anticancer efficacy of PD-1/PD-L1 blockade via combination therapy and PD-L1 regulation

**DOI:** 10.1186/s13045-022-01242-2

**Published:** 2022-03-12

**Authors:** Mengling Wu, Qianrui Huang, Yao Xie, Xuyi Wu, Hongbo Ma, Yiwen Zhang, Yong Xia

**Affiliations:** 1 Department of Rehabilitation Medicine, State Key Laboratory of Biotherapy and Cancer Center, National Clinical Research Center for Geriatrics, West China Hospital, Sichuan University, Chengdu, 610041 China; 2 Department of Obstetrics and Gynaecology, Sichuan Provincial People’s Hospital, University of Electronic Science and Technology of China, Chengdu, 610072 China; 3 Chinese Academy of Sciences Sichuan Translational Medicine Research Hospital, Chengdu, 610072 China; 4 Key Laboratory of Rehabilitation Medicine in Sichuan Province/Rehabilitation Medicine Research Institute, Chengdu, 610041 China

**Keywords:** Immunotherapy, PD-1/PD-L1, Combination therapy, PD-L1 regulation

## Abstract

Immune checkpoint molecules are promising anticancer targets, among which therapeutic antibodies targeting the PD-1/PD-L1 pathway have been widely applied to cancer treatment in clinical practice and have great potential. However, this treatment is greatly limited by its low response rates in certain cancers, lack of known biomarkers, immune-related toxicity, innate and acquired drug resistance, etc. Overcoming these limitations would significantly expand the anticancer applications of PD-1/PD-L1 blockade and improve the response rate and survival time of cancer patients. In the present review, we first illustrate the biological mechanisms of the PD-1/PD-L1 immune checkpoints and their role in the healthy immune system as well as in the tumor microenvironment (TME). The PD-1/PD-L1 pathway inhibits the anticancer effect of T cells in the TME, which in turn regulates the expression levels of PD-1 and PD-L1 through multiple mechanisms. Several strategies have been proposed to solve the limitations of anti-PD-1/PD-L1 treatment, including combination therapy with other standard treatments, such as chemotherapy, radiotherapy, targeted therapy, anti-angiogenic therapy, other immunotherapies and even diet control. Downregulation of PD-L1 expression in the TME via pharmacological or gene regulation methods improves the efficacy of anti-PD-1/PD-L1 treatment. Surprisingly, recent preclinical studies have shown that upregulation of PD-L1 in the TME also improves the response and efficacy of immune checkpoint blockade. Immunotherapy is a promising anticancer strategy that provides novel insight into clinical applications. This review aims to guide the development of more effective and less toxic anti-PD-1/PD-L1 immunotherapies.

## Introduction

Immunotherapy, a promising anticancer strategy that improves the specificity and strength of the immune response to cancer, has been widely studied in recent years. Brakes on the immune system protect healthy tissues and organs from attack by the immune system; this brake system is hijacked by cancer cells to escape from the immune system or even turn against it [1]. The programmed cell death 1 receptor (PD-1)/programmed cell death ligand 1 (PD-L1) pathway and the cytotoxic T-lymphocyte-associated protein 4 (CTLA-4) pathway constitute the well-known brake system of the immune system. Targeting these two pathways has been shown to be a successful anticancer strategy [2]. Antibodies against the PD-1/PD-L1 pathway have been extensively applied to cases of melanoma, lung cancer, lymphoma, liver cancer, colorectal cancer, urothelial cancer, squamous cell carcinoma of the head and neck, cervical cancer, kidney cancer, stomach cancer and breast cancer [3]. This monotherapy or combination therapy (as adjuvants or neo-adjuvants) produces a remarkable clinical response. A small number of cancer patients subsequently experience long-term remission. Nevertheless, the PD-1/PD-L1 blockade, similar to other anticancer treatments, is also limited by a low response rate in certain cancers, lack of known biomarkers, immune-related toxicity and innate and acquired drug resistance. To date, the clinical response to PD-1/PD-L1 blockade is barely 40% [4]. Thus, identifying optimal biomarkers for screening cancer patients who are responsive to immune checkpoint blockades (ICBs) and accurately monitoring its therapeutic efficacy is of great clinical importance [5]. In addition, it is important to precisely distinguish cancer cells from normal cells in ICBs, thus preventing severe adverse events such as discontinued treatment, dose reduction or even death due to immune-related toxicity [6]. Similar to other anticancer treatments, some patients may not be sensitive to ICB or develop drug resistance after a period of medication. Elucidating the potential mechanisms of low responses and drug resistance to ICB will enhance their clinical benefits [7] and is key to improving the efficacy of immunotherapy [8].

In the present review, we first illustrate the biological mechanisms of PD-1/PD-L1 immune checkpoints and their role in both the normal immune system and TME, aiming to enhance current understanding of the immune checkpoint molecules PD-1/PD-L1. Combination therapy with other standard treatments, such as chemotherapy, radiotherapy, targeted therapy, anti-angiogenic therapy, other immunotherapies and even diet control, is expected to address the limitations of PD-1/PD-L1 blockade. Either upregulation or downregulation of PD-L1 expression in the TME improves the therapeutic efficacy of ICBs; a combination therapy of either with immunotherapy may represent a novel anticancer treatment and combinatorial drug design. This review summarizes the latest developments, prospects and challenges of the combination therapy of PD-1/PD-L1 blockade and PD-L1 regulation, aiming to provide novel ideas for developing more effective and less toxic anti-PD-1/PD-L1 immunotherapy.

## Immune checkpoints in cancer therapy

### The immune system in carcinogenesis

Advanced cancer has mainly been treated with radiotherapy and chemotherapy in recent decades. However, these treatments are unable to distinguish normal cells from cancer cells, leading to damage of normal cells, severe adverse events and even discontinuation of treatment. The normally functioning immune system is capable of accurately recognizing and eliminating cancer cells due to significant differences between normal cells and cancer cells, thus achieving precision killing. The interaction between cancer cells and the immune system used to be considered the main determinant factor for carcinogenesis [9].

However, recent evidence has shown that most new tumors formed in the esophagus would naturally be eliminated due to the weaker viabilities of these newly formed tumors that of adjacent mutant epithelial cells, rather than differences in survival due to the involvement of the immune system [10]. Mutations are the potential origin of cancers. It was recently found that carcinogenicity is mediated by oncogenes (e.g., BRAFV600E), lineage-specific transcription factors (e.g., SOX10) and chromatin factors for regulating development (e.g., ATAD2) [11].

A recent study analyzed the relationship between immune response and tumor development [12], finding that chronic inflammatory cells secrete IL-6 and that transient inflammation leads to persistent reprogramming of epithelial cells leading to subsequent tumorigenesis, thus underscoring the role of the immune system in promoting tumorigenesis. Established anti-tumor immune responses suppress tumor development, but tumor cell clones that escape immune surveillance eventually develop into clinically visible tumors.

Cancer immunotherapy eliminates cancer cells by stimulating and enhancing immune function or regulating the immune state based on immune surveillance and immune editing. Of all immune cells, T cells are the most powerful tool for directly killing cancer cells and are characterized by high specificity, strong memory and high adaptability [13]. The cancer-immunity cycle, in which cancer cells release specific antigens and the immune system is activated to kill them, is a cyclical process involving 7 steps: (1) Antigens are expressed and released by cancer cells; (2) cancer antigen processing and presentation; (3) T cell initiation and activation; (4) T cell migration to cancer lesions; (5) T cell penetration to cancer lesions; (6) recognition of cancer cells by T cells; and (7) elimination of cancer cells by T cells [14]. Multiple factors in this cancer-immunity cycle are potential therapeutic targets for immunotherapies. Cancer cells have been reported to express high levels of immunosuppressive signal proteins, which contribute to avoid the attack of immune cells in the TME.

### The basic biology of immune checkpoints

T cells are the most important part of the immune system, and their function is strictly and precisely regulated by the immune system, as multiple receptor molecules on the cell membrane transduce activating or inhibitory signals. Once T cells are activated by antigen stimulation, the immune system also initiates negative feedback to avoid continuous overactivation of T cells that causes excessive damage to the body. Inhibitory receptor molecules, known as checkpoint molecules, expressed on the surface of T cells are responsible for the negative feedback of the immune system, inhibiting the elimination of target cells by T cells by binding corresponding ligand molecules on the target cell surface. Checkpoint molecules are well studied in translational research in immunotherapies [15].

Immune checkpoint inhibitors (ICIs) have recently been highlighted for their functions in blocking the effect of inhibitory immune molecules on T cells and thus reducing immune tolerance to cancers; these ICIs have been widely analyzed by biopharmaceutical companies. Many immune checkpoints have been identified, including CTLA-4 and PD-1; while both have been thoroughly investigated, PD-1 has been of particular interest and has been widely applied in clinical practice.

## The PD-1/PD-L1 pathway in cancer immunotherapy

### PD-1/PD-L1 structure

PD-1 is a cell surface receptor that was initially found to be preferentially expressed in apoptotic cells [16]. Later, PD-1 was identified as the key immune checkpoint for regulating T and B cell response thresholds to antigens. As a key checkpoint for T cells, PD-1 exerts a central role in regulating their cellular functions. The interaction between PD-L1 and PD-1 inhibits T cell function by inducing T cell exhaustion to promote immune evasion [17]. Therefore, abnormally upregulated PD-L1 levels in cancer cells and some immune cells results in immune escape. Anti-PD-1/PD-L1 antibodies have become a hot topic in cancer immunotherapy.

PD-1, also known as CD279, is a type I transmembrane protein encoded by the *PDCD1* gene of the CD28 immunoglobulin superfamily. It was first discovered and reported by Ishida et al. in 1992 [15, 16]. PD-1 is mainly expressed in activated CD4^+^ T cells, CD8^+^ T cells, natural killer T cells, B cells, macrophages, dendritic cells (DCs) and monocytes; its expression is induced by the T or B cell receptor pathway and enhanced by the stimulation of tumor necrosis factor [18]. However, naive T and B cells barely express PD-1 [19–21]. PD-1 is comprised of 288 amino acids, including a single Ig variable-type (IgV) extracellular domain, a transmembrane domain and a cytoplasmic domain [22–24]. Its extracellular domain is similar to that of other members of the CD28 superfamily, containing an Ig variable-type domain that is important in ligand binding. N-terminal and C-terminal tyrosine residues in the cytoplasmic domain are involved in the formation of immunoreceptor tyrosine-based inhibitory motifs (ITIMs) and immunoreceptor tyrosine-based switch motifs (ITSMs), respectively [16, 24–26]; the latter is the main signal transduction domain of PD-1 and is closely related to the response activity of effector T cells.

The biological functions of PD-1 rely on two ligands: PD-L1 (also known as B7-H1 or CD274) and PD-L2 (also known as B7-H2 or CD273). The former was initially discovered by Dong et al. in 1999 [27], and the latter was discovered by Tseng et al. [28]. PD-L1 is widely expressed in T cells, B cells, DCs, cancer cells, macrophages and others and is further upregulated by activated proinflammatory cytokines [29]. It is mainly responsible for the immune escape of cancers.

### The role of PD-1/PD-L1 in the immune system and in cancers

Under normal circumstances, the PD-1/PD-L1 pathway negatively regulates the immune system. ITSMs are a vital site for the biological functions of PD-1, which is phosphorylated by binding to PD-L1 and further induces immune inhibition by activating a series of intracellular pathways [3]. Notably, the specific mechanisms by which PD-1 exerts its immunosuppressive effects differs between T and B lymphocytes [30].

Two signal pathways are involved in the immune response induced by T cells following pathogen invasion: the binding of major histocompatibility complexes (MHCs) on the antigen presenting cell (APC) surface to T cell receptors (TCRs) and the binding of APC-expressed immunostimulatory ligands to TCRs. As a result, activating or inhibitory signals are transduced to T cells and further regulate immune responses, such as T cell activation and exhaustion. PD-1/PD-L1 pathway can inhibit TCR-mediated T cell activation. In T cells, the engagement of PD-1 ligands and PD-1 results in the recruitment of SHP-1/2 (Src homology 2-containing tyrosine phosphatase 1/2) to the C-terminal of the ITSM. SHP-2 then dephosphorylates TCR-associated CD-3ζ and ZAP70, resulting in the inhibition of downstream signaling [31]. Specifically, phosphatidylinositol 3-kinase (PI3K) pathway is suppressed, and the expression of the cell survival gene Bcl-XL is reduced [32]. In addition, PD-1 inhibits TCR-induced activation of the PI3K/AKT pathway by activating PTEN [33]. Moreover, by inhibiting the activation of the RAS-MEK-ERK pathway, PD-1 suppresses the proliferation of T cells [34]. PD-1 has been reported to inhibit the activation of PKCδ, thereby decreasing the level of cytokine secreted by T cells, such as IFN-γ and IL-2 [35]. Furthermore, PD-1 signaling regulates T cell metabolism by suppressing glycolysis and promoting lipolysis and fatty acid oxidation [36].

PD-1/PD-L1 interaction also inhibits the activation of B cells. When PD-L1 binds to PD-1, two tyrosines on its ITSM bind to the B cell receptor (BCR) and are phosphorylated, which recruits SHP-2 to the C-terminus of PD-1; SHP-2 is then phosphorylated. Subsequently, phosphorylated SHP-2 dephosphorylates the BCR, thus leading to acute Ca^2+^ disorder and long-term growth arrest. Therefore, PD-1 can impair the immune response of B cells to antigens [35].

The brake system of PD-1/PD-L1 was gradually developed during the course of evolution. In this brake system, negative feedback terminates the killing effect of the immune system in a timely manner and thus protects against excessive damage to normal tissues. Generally, the PD-1/PD-L1 pathway prevents the overstimulation of T cells and maintains immune tolerance to self-antigens, thereby reducing damage to surrounding tissues and preventing autoimmune diseases from developing [37, 38].

Cunningly, cancer cells escape the killing effect induced by T cells by utilizing this brake system. Overexpression of PD-L1 induces the development of an immunosuppressive TME in multiple cancers [39–41], including non-small-cell lung cancer (NSCLC) [42, 43], melanoma [44], renal cell carcinoma (RCC) [45], prostate cancer [46], breast cancer [47] and glioma [48].

Cancer cells highly express PD-L1 on the cell membrane. The binding of PD-L1 to PD-1 in T cells produces negative signals, inducing T cell apoptosis and reducing immunocompetence, which thus helps cancer cells escape immune surveillance and killing. In addition, the activation of the PD-1/PD-L1 pathway negatively affects the differentiation of effector T cells (Teff) and memory T cells (Tm) and upregulates the differentiation of regulatory T cells (Treg) and exhausted T cells (Tex), thereby significantly inhibiting the immune effect of T cells [49]. The binding of PD-L1 to PD-1 also inhibits the proliferation of tumor-specific T cells and induces apoptosis by triggering the release of cytokines and cytotoxins [50]. Cancer cells are also able to transport PD-L1 (carried in exosomes) to remote regions via the circulatory system. Therefore, they can remotely inhibit T cell activity before reaching metastatic lesions [51–53].

**Fig. 1:**
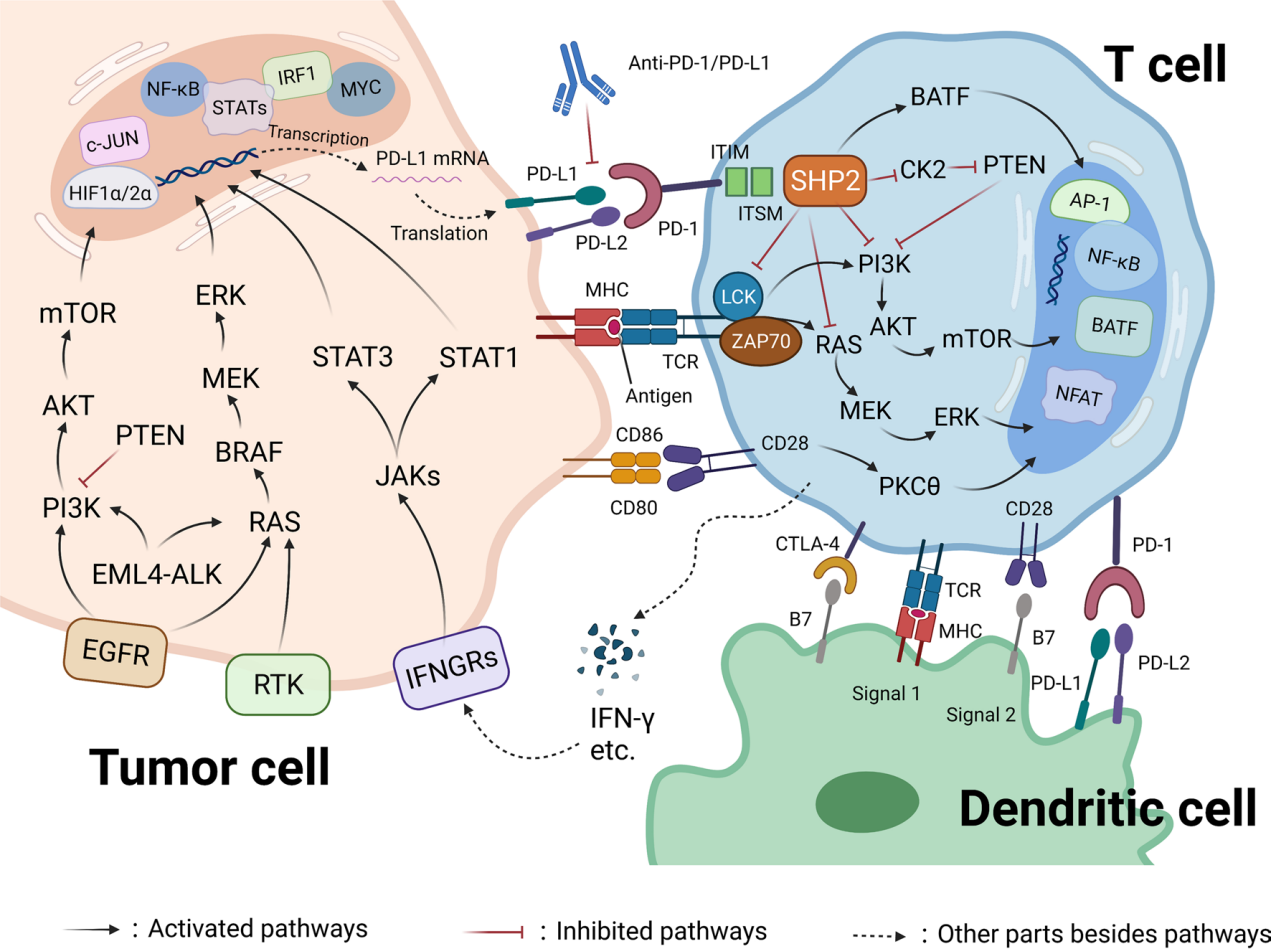
PD-1/PD-L1 interaction mediated T cell inhibition. Factors that regulate PD-L1 expression mainly includes (1) genomic aberrations, (2) microRNA-based control, (3) oncogenic transcription factors and pathways and (4) posttranslational regulation and transport. The RAS/MEK/ERK, PI3K/Akt/mTOR, JAK/STATs signaling and TLRs/IKKs pathways are the main pathways regulating PD-L1 expression. IRF1, STATs, MYC, NF-κB, c-Jun and HIF1α/2α are the main downstream transcription factors. Posttranslational modifications of PD-L1 include phosphorylation, ubiquitination, glycosylation and palmitoylation. Induction of PD-L1 by cytokines, such as IFN-γ, is considered a secondary mechanism. Activation of PD-1/PD-L1 signaling leads to the recruitment of the phosphatase SHP-2 to the C-terminal of the ITSM, which downregulates the RAS-MEK-ERK and PI3K-Akt-mTOR pathways and attenuates LCK-induced phosphorylation of ZAP70. In addition, SHP-2 induces the expression of BATF, which inhibits the expression of some effector genes. In general, activation of PD-1/PD-L1 signaling leads to the inhibition of T cell proliferation and activation. Activation of PD-1/PD-L1 can be blocked by anti-PD-1/PD-L1 antibodies. In addition, APCs uptake tumor antigens and regulate T cell responses through the interaction between major MHC and TCRs. APCs (dendritic cells) regulate T cell activity through modulating the interaction between PD-L1/PD-L2 and PD-1 and the interaction between B7 and CD28. CTLA-4 is a negative regulator of costimulation that is activated in the recognition of specific tumor antigens presented by APCs

Blocking the binding of PD-L1 to PD-1 blocks this negative feedback and restores the function of T cells as well as their ability to kill cancer cells. Therefore, ICIs (PD-1/PD-L1 inhibitors) exert their anticancer effect via the immune system of the host, which is quite different from conventional cancer therapies. To date, the extraordinary efficacy of ICIs has been validated in multiple types of solid tumor cancers and hematological malignancies, with a sustained response and long-term survival benefits [54–58]. The inhibitory effect of PD-1/PD-L1 pathway on T cells is shown in Fig. 1.

### The regulation of PD-1/PD-L1 in the TME

The TME is mainly composed of tumor-associated stromal cells, extracellular matrix (ECM) [59], nonmalignant cells surrounding malignant lesions and complex signaling networks for maintaining the internal interactions of the TME [60]. TMEs not only promote the growth of cancer cells but also trigger invasion and metastasis [61]. In addition, exosomes carrying noncoding RNAs are vital components of the TME and provide favorable conditions for the growth and migration of cancer cells [62].

The ECM is comprised of the basement membranes and interstitial matrix [63], an important barrier for metastasis. Various substances can be found in the ECM, including a large number of growth factors, cytokines and metalloproteinases secreted by cancer cells and other cells in the TME, as well as acidic substances produced by cancer metabolism. These acidic substances in turn maintain the weakly acidic TME, induce epithelial-to-mesenchymal transition (EMT) and promote the formation of a hypoxic microenvironment.

#### Regulatory effects of the TME on PD-1

The influence of PD-1 on immune suppression is very complicated. Previous studies have shown that PD-L1 expressed in cancer cells induces immune suppression through the apoptosis of activated T cells and the production of IL-10 by stimulated T cells [27]. Furthermore, persistent activation of PD-1 decreases glucose metabolism in T cells and induces T cell incompetence and exhaustion [64–66]. In a chronic lymphocytic choriomeningitis virus (LCMV) infection mouse model, persistent antigen exposure exhausted T cells and completely or partially eliminated effector T cell function, which was reversed by the application of an anti-PD-L1 antibody [67]. In addition to inducing cell differentiation into Tregs, PD-1 also regulates their development and cellular functions [68].

When stimulated by inflammatory factors, DCs upregulate PD-1 and thus significantly inhibit the antibacterial ability of the innate immune system [69]. PD-L1 on the surface of macrophages regulates T cell migration, leading to the active immune expulsion of T cells from the TME [70]. In addition to regulating immune tolerance, PD-L1 expressed in cancer cells quickly establishes a molecular barrier to fight against the killing effect of immune effector cells [71]. By regulating the mTOR pathway, PD-1 expressed in melanoma cells promotes malignant growth [72].

As an immunosuppressive molecule, PD-1 inhibits the activation of T cells and induces their apoptosis. PD-1 is expressed at low levels in naive T lymphocytes and can be immediately activated by TCRs. Transforming growth factor β (TGF-β) is highly important in the process of PD-1 activation by TCRs [73]. It induces abundant expression of PD-1 on the immune cell membrane following antigen stimulation, which may be attributed to a self-protection mechanism that prevents the overactivation of immune cells.

Other factors in the TME can also regulate the expression level of PD-1. IL-7, IL-15 and IL-21 can induce the activation of PD-1 in peripheral T lymphocytes [74]. Although upregulated PD-1 does not affect the expansion and survival of T cells, it inhibits the secretion of cytokines [75]. IL-12 and IL-6 induce PD-1 during the activation of TCRs by altering the chromatin structure of the PD-1 gene and activating the STAT3/STAT4 pathway, in which the proximal cis-acting elements in the promoter region of PD-1 and the transcription factors FOXO1 and NF-κB are needed [76]. Moreover, the inflammatory factors TNF-α and IL-6 regulate the growth inhibition of T cells in osteoarthritis by blocking the interaction between PD-1 and PD-L1 via induction of the secretion of soluble PD-1 [77]. In macrophages, interferon-α (IFN-α) regulates the expression of PD-1 by activating the JAK/STAT pathway. The interferon-sensitive responsive element (ISRE) in the promoter region of PD-1 enhances PD-1 transcription by forming the p48/STAT1/STAT2 complex with the JAK/STAT pathway [78]. Additionally, IFN-α has been reported to synergistically regulate the expression of PD-1 with TCRs, producing a strong inhibitory feedback signal targeting the T cell-induced immune response [79].

#### Regulatory effects of the TME on PD-L1

PD-L1 causes T cell exhaustion and immune tolerance, which is the main factor for the immune escape of cancer cells [80, 81]. In addition to expression on the cell surface of T lymphocytes, B lymphocytes, DCs and macrophages, PD-L1 is also highly expressed on the surface of cancer cells. A variety of cytokines and exosomes in the TME inhibit the activation of cytotoxic lymphocytes (CTLs) by inducing the expression of PD-L1 and activating the PD-1/PD-L1 pathway, which eventually promote immune escape. The main regulatory factors for PD-L1 are reviewed below.

##### Interferon-γ

Interferon is a biologically active glycoprotein secreted following viral infections; it has antiviral, antibacterial, antitumor and immunomodulatory functions [82]. Interferon-γ (IFN-γ) is a type II IFN that is mainly secreted by CD8^+^ T lymphocytes, NK cells and macrophages. IFN-γ can promote cancer growth and resist immune surveillance in certain circumstances [83]. An increasing number of studies have validated the induction of cancer progression by IFN-γ through activation of PD-L1 and immune escape from the attack of certain subtypes of T cells [35, 84]. IFN-γ induces the expression of PD-L1 through multiple pathways, and analyzing these pathways facilitates the development of novel cancer therapies with fewer adverse events.

##### Tumor necrosis factor-α

As an inflammatory cytokine, tumor necrosis factor-α (TNF-α) activates inflammatory cells, kills pathogens, stimulates tissue repair and induces angiogenesis and connective tissue formation. However, it facilitates the immune escape of tumor cells by upregulating expression of PD-L1 [85]. TNF-α is mainly produced by activated macrophages, T cells and NK cells, which bind to specific homotrimeric receptors on the cell membrane. By activating the NF-κB and ERK1/2 pathways, TNF-α upregulates PD-L1 expression at both the mRNA and protein levels [85]. In addition, it stimulates cell growth, differentiation and apoptosis by inducing an inflammatory response through the activation of caspase, JNK and NF-κB. TNF-α also regulates the expression of PD-L1 by targeting miRNA-155 [86].

##### Interleukins

Interleukins (ILs) are a type of cytokine that are important in the maturation, activation, proliferation and immune regulation of immune cells and participate in multiple physiological and pathological processes. The proinflammatory cytokines IL-6 and IL-17 regulate the expression of PD-L1 in the TME. Epithelial growth factor receptor (EGFR) regulates the expression of PD-L1 as well as cell proliferation through the IL-6/JAK/STAT3 pathway [35, 73, 76–80, 82–85, 87]. In addition, overexpression of PD-L1 and knockdown of NKG2D enhance NSCLC patient tolerance of radiotherapy through the IL-6/MEK/ERK pathway [88]. During carcinogenesis, IL-6 interacts with proteins involved in the formation of proliferative matrix and drives myeloid suppressor cells, thereby suppressing the immune system. Therefore, inhibiting the IL-6 pathway in the TME can enhance the cytotoxic response and sensitivity of cancer cells to NK cells by downregulating PD-L1 expression [89, 90].

##### Epithelial growth factor

Epithelial growth factor (EGF) is a small-molecule active peptide widely distributed in the human body. EGF contributes to cell growth by binding to corresponding receptors and activating the EGFR pathway. The EGFR pathway is well known for its regulation of cancer cell migration and proliferation. Moreover, EGFR mutations that trigger malignant proliferation and metastasis without the need to bind to EGF have been detected in many types of cancer cells. The EGFR pathway has also been reported to be involved in immune escape. EGF upregulates PD-L1 expression in lung cancer, breast cancer, head and neck cancer, esophageal cancer and salivary adenoid cystic carcinoma. MYC, an important transcription factor in cancers, is also involved in the regulation of PD-L1 by EGFR. In the EGFR-derived PD-L1 pathway, knockdown of MYC significantly downregulates PD-L1 expression [91–93]. MYC upregulates PD-L1 expression in T cell acute lymphoblastic leukemia (T-ALL) cells by directly binding to the promoter region of PD-L1, suggesting that the EGFR pathway is able to upregulate PD-L1 by upregulating MYC and promoting nuclear translocation [94]. EGF not only induces the transcription of PD-L1 but also influences its protein stability and biological function. Additionally, the RAS-EGFR pathway is a classic oncogenic intracellular pathway that promotes tumor immunoreactivity by regulating the mRNA stability of PD-L1 [95]. A previous study reported that K-RAS mutations in EGFR-driven lung cancer were associated with the expression of PD-L1 [96, 97].

##### Exosomes

Exosomes are extracellular vesicles (40–150 nm in diameter) released by almost all types of cells. They serve to transduce intracellular information to other cells and thus change their activity [98, 99]. Functionally, exosomes can regulate the growth, migration and angiogenesis of cancer cells [62, 100]. Cancer-derived exosomes can promote macrophage polarization into M2 macrophages and the expression of PD-L1 in these macrophages by upregulating phosphorylated STAT3 and further enhancing the immunosuppressive effect [101]. Consistent with these findings, cancer-derived exosomes containing PD-L1 have been found to have a strong immunosuppressive effect [51]. Cancer-derived exosomes in chronic lymphocytic leukemia induced an immunosuppressive response in monocytes. Monocyte activation is mainly induced by noncoding microRNAs contained in exosomes which activate the TLR7 pathway in monocytes, promoting monocyte-induced secretion of cytokines and eventually inducing the expression of PD-L1 [102]. At present, research on exosomes is in its infancy, and we believe that the novel regulatory effects of exosomes on PD-L1 will be elucidated in the future.

## The PD-L2 pathway in cancer immunotherapy

### Molecular structure and distribution of PD-L2

PD-L2, also known as B7-DC, CD273 or PDCD1LG2, is the second most important ligand for binding to PD-1after PD-L1 [103]. PD-L2 protein is a type I transmembrane protein encoded by the *PDCD1LG2* gene, consisting of 270 amino acid residues and located on chromosome 9 with PD-L1 [104]. The extracellular domain of PD-L2 consists of a membrane-distal immunoglobulin variable region and a membrane-proximal immunoglobulin constant region [22]. Several studies have shown that the affinity of the PD-L2/PD-1 interaction is 3–4 times higher than that of the PD-L1/PD-1 interaction. This difference in affinity is attributed to the presence of a tryptophan residue unique to PD-L2 that binds to a binding site on the surface of PD-1 [105]. PD-L2 is expressed primarily by dendritic cells, macrophages and cancer cells and downregulates the effector functions of T cells through the PD-1/PD-L2 axis in the TME [106]. PD-L2 is expressed on activated CD4^+^ T cells and CD8^+^ T cell subsets, which can bind to PD-1 on T cells and inhibit T cell activation and proliferation [107].

### PD-1/PD-L2 axis–mediated immune escape in cancer

The PD-1/PD-L2 pathway plays a major and complex role in the development and progression of cancer. The regulatory role of PD-L2 on T cells in the TME has been controversial. Some studies have shown that PD-L2 suppresses immune function by binding to PD-1 co-inhibitory receptors [103, 108]. However, other studies have shown that PD-L2 is a positive co-stimulatory molecule that stimulates T cell proliferation and cytokine production, exerting its functions through receptors other than PD-1 [28, 109]. In human T cells, PD-L2 acts only as a negative regulator of T cell activity, inhibiting T cell proliferation by interacting with PD-1, reducing cytokine production and leading to cell-cycle arrest [103, 107, 110]. Cancer cells frequently achieve immune escape through the PD-1/PD-L2 pathway mediated by potent inhibitory signals, thereby hindering the proliferation and function of effector T cells and forming an immune escape microenvironment that suppresses anti-tumor immunity [29, 111–113]. Tumors can induce immune escape via various mechanisms, thereby evading cytotoxicity from the immune system, and eventually progress and metastasize to other parts of the body [112, 113]. During tumorigenesis, the PD-1/PD-L2 signaling pathway can cause the exhaustion of T cell function and promote immune escape. The inhibitory effect of PD-L2 on T cell function involves the regulation of the PI3K/AKT and MEK/ERK pathways [110, 114]. T cell activity is regulated not only by receptor tyrosine kinases (RTKs) but also by non-RTKs. Studies on SHP-1 and SHP-2 have shown that they regulate T cell activity [115, 116]. PD-L2 inhibits the PI3K/AKT and MAPK pathways while also increasing the phosphatase activity of SHP-2 [110]. PD-1/PD-L2-induced SHP-2 activation is involved in the early signaling pathways required for negative regulation of T cell function, such as cytokine production, and cell adhesion [110]. Concomitant with T cell receptor (TCR) or B-cell receptor (BCR) cross-linking, PD-1 binds to PD-L2 and induces inhibitory signals by recruiting phosphatases (e.g., SHP-2) to the ITSM in the cytoplasmic tail of PD-1, resulting in the dephosphorylation of effector molecules involved in downstream TCR or BCR signaling[117].

## Advances in PD-1/PD-L1 blockade-based combination treatment for cancer

As an adjuvant therapy, immunotherapy has become the next focus of competition in the clinical development of anticancer drugs. Immunotherapy has exhibited positive results both as adjuvant or neoadjuvant therapy in the clinical treatment of cancer. Since this review focuses on PD-1/PD-L1 blockade, immunotherapy and ICB mentioned later refer to PD-1/PD-L1 blockade, and ICI refers to PD-1/PD-L1 inhibitor.

Although great progress has been made in immunotherapy of cancer, they face challenges in cancer therapy mainly due to their low response rate. Atezolizumab is a human-derived anti-PD-L1 inhibitor approved by the Food and Drug Administration (FDA) in 2016 for the treatment of urothelial cancer. The clinical trial IMVigor 210 reported that the target response rate in patients with metastatic urothelial cancer expressing moderate to high levels of PD-L1 was only 27%. The PD-L1 inhibitor does not exhibit its predicted effects in up to 73% of patients with high levels of PD-L1, probably due to innate resistance [118, 119]. Unfortunately, this low level of therapeutic efficacy may reduce even further in patients responsive to ICB after a long-term treatment, a phenomenon known as acquired resistance. Most melanoma patients with a good response to ICB have been reported to only experience limited or transient benefits of ICB treatment [4, 9]. Although clinical evidence has supported the role of immune surveillance in controlling the recurrence and progression of some common types of cancers, such as prostate cancer, ovarian cancer, breast cancer and colorectal cancer with microsatellite instability-high (MSI-H) [13, 120], most patients have difficulty benefiting from ICB [9, 121–123]. To date, the molecular mechanisms underlying acquired resistance remain unclear, which significantly hinders the sustainability of ICB treatments. Hodgkin's lymphoma and melanoma have the best response to ICB, while head and neck squamous cell carcinoma and gastrointestinal cancers do not show high response rates. The response rate of NSCLC is medium, but the resistance rate remains high. In general, the following five mechanisms are thought to explain acquired resistance to ICB. First, tumor antigen presentation may be damaged due to the downregulation of MHC class I molecules or deficiency in antigen presentation induction; as a result, TCRs would be unable to recognize tumor antigens, and ICB would become invalid. Second, IFN-γ sensitivity may be lost. IFN-γ activates the JAK-STAT pathway, which upregulates MHC class I molecules and enhances anticancer immunity. However, inactivating mutations of JAK1 and JAK2 occur during ICI treatment, which would eliminate the sensitivity of cancer cells to IFN-γ. Third, neoantigens may be eliminated. Selective pressure in the TME during anti-PD-1 treatment may clear the neoantigen without the production of neoantigen-specific T cells, and therefore, immune escape could develop. Fourth, cancer-induced immune inhibition could occur. Stabilized β-catenin induced by WNT and the loss or mutation of PTEN facilitates the production of inhibitory cytokines, which would further prevent the infiltration of CD8^+^ T cells and inhibit their functions. Fifth, positive expression of other ICIs may occur. Several other immune checkpoints are produced during a single ICI treatment, thus resulting in acquired drug resistance [124–127].

Most conventional anticancer therapies also lead to drug resistance; however, combination with ICB may produce a satisfactory outcome by overcoming drug resistance. Combination therapy aiming to enhance anticancer efficacy is of major interest. ICB can be combined with chemotherapy, radiotherapy, surgery, targeted therapy and antiangiogenic therapy. Combination therapy with ICB not only enhances the capacity of antigen presentation and rescues exhausted effector T cells but also activates the immune system by releasing cancer antigens and stimulating them to kill cancer cells, which may yield enhanced anticancer efficacy [128]. In addition, changes in killing factors and immune factors that attack tumor cells potentially influence immunotherapy efficacy.

Nevertheless, combination therapy is significantly restricted by the occurrence of severe adverse events (AEs). A recent systematic review and meta-analysis reported that the incidence of treatment-related AEs in combination therapy with chemotherapy and anti-PD-1/anti-PD-L1 antibodies was up to 97.7%, which is the highest of all types of combination therapies for cancers [129]. This meta-analysis included 36 clinical trials with 43 regimens. Among them, the incidence of all-grade treatment-related AEs was 97.7%, and the most common AEs were anemia (45.4%) and hair loss (45.1%). The incidence of grade 3 and above AEs was 68.3%, and the most common AEs were neutropenia (19.6%) and anemia (11.4%) [129].

It also analyzed combination therapy with immunotherapy and targeted therapy in 45 clinical trials with 47 regimens. The incidence of all-grade treatment-related AEs was 94.5%, and the most common AEs were fatigue (34.3%) and diarrhea (31.7%). The incidence of grade 3 and above AEs was 68.3%, and the most common AEs were hypertension (9.3%) and hyponatremia (3.6%) [129].

Additionally, combination therapy with different immunotherapies was analyzed, including 54 clinical trials with 57 regimens. The incidence of all-grade treatment-related AEs was 86.8%, and the most common AEs were fatigue (26.4%) and diarrhea (21.1%). The incidence of grade 3 and above AEs was 35.9%, and the most common AEs were lipase increase (7.2%) and colitis (3.6%) [129].

Moreover, the meta-analysis analyzed combination therapy with immunotherapy and radiotherapy in 7 clinical trials with 7 regimens. The incidence of all-grade treatment-related AEs was 89.4%, and the most common AEs were dysphagia (30.3%) and nausea (24.9%). The incidence of grade 3 and above AEs was 12.4%, and the most common AEs were lymphocytopenia (10.3%) and dysphagia (8.8%) [129].

It is urgent to develop more reasonable combination therapies with fewer AEs and higher survival benefits. In the following section, we mainly summarize combination therapy with ICIs and others in NSCLC patients. ICB has achieved unprecedented efficacy in the treatment of NSCLC patients. However, only a small number of NSCLC patients have exhibited high response to ICB [121].

**Fig. 2:**
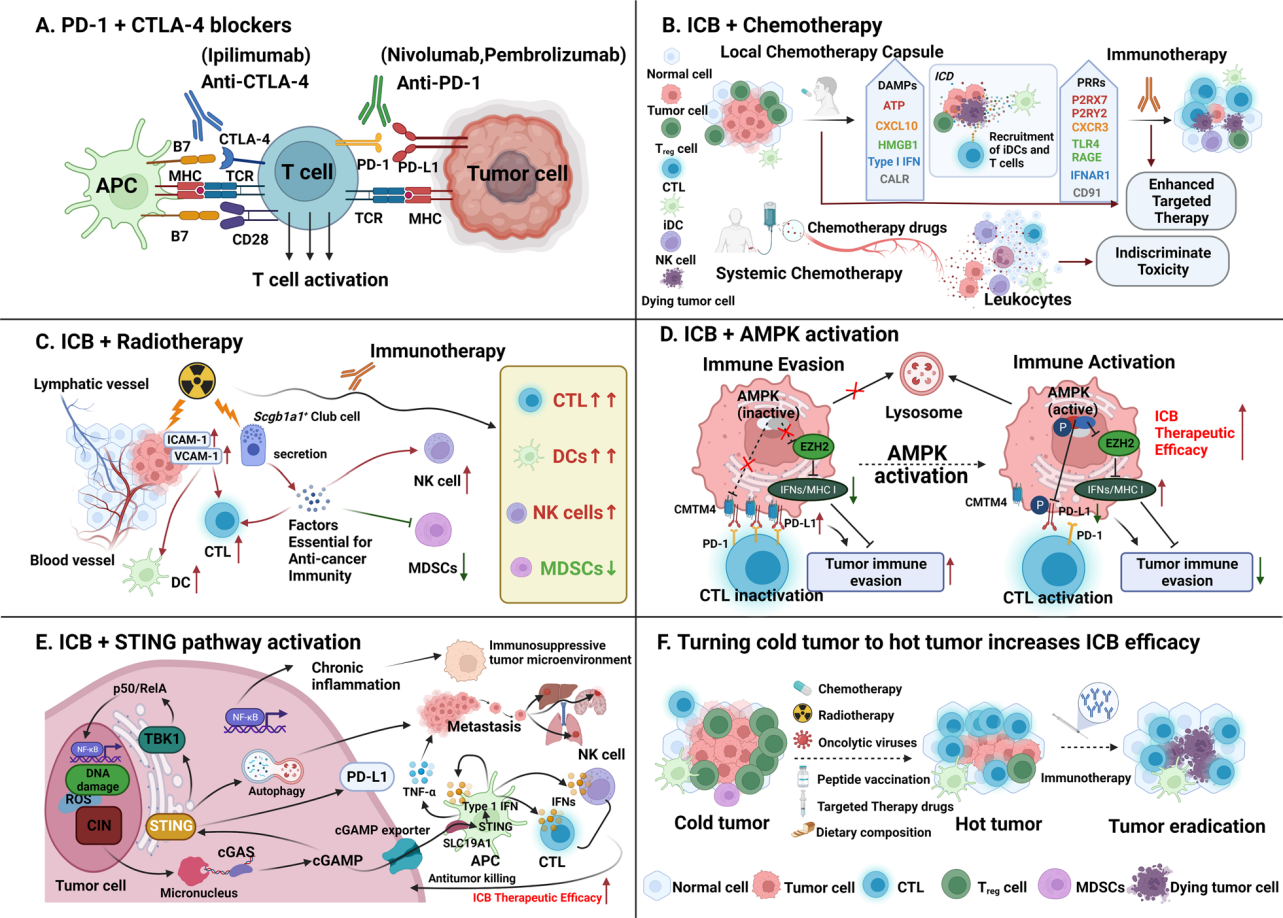
Combination strategies to enhance the therapeutic efficacy of PD-1/PD-L1 blockade. **A** Combination therapy with PD-1 and CTLA-4 blockers. The activation of PD-1/PD-L1 and CTLA-4 can be blocked by anti-PD-1/PD-L1 and CTLA-4 antibodies, respectively. The combined application of PD-1 and CTLA-4 inhibitors produces synergistic effects. **B** Combination therapy with chemotherapy. Chemotherapy is able to induce ICD, promote the release of tumor antigens and DAMPs, activate DCs, induce local production of CXCL10, recruit T cells to the tumor bed and enhance the differentiation of antitumor-specific CTLs. Chemotherapy can also reduce the number of immunosuppressive cells, such as MDSCs and Tregs. However, systemic chemotherapy shows undifferentiated toxicity to tumor cells and the anticancer immune system, while local chemotherapy enhances immunotherapy by remodeling the TME and attracting activated immune cells to the tumor region. **C** Combination therapy with radiotherapy. Radiotherapy markedly upregulates the cell adhesion factors ICAM-1 and VCAM-1 on the surface of cancer cells. One of the mechanisms by which radiotherapy may enhance immunotherapy is through activation of certain types of club cells which release proteins that are beneficial to immunotherapy. **D** Combination therapy with an AMPK activator. Reduced PD-L1 levels in the presence of AMPK activation could enhance the efficacy of combining ICB with an AMPK activator. **E** Combination therapy with STING agonists. The cGAS-STING pathway is essential for linking the innate immunity and adaptive immunity against cancers. Cancer cells can escape immune surveillance by inactivating the cGAS-STING pathway. Therefore, ICB can be combined with STING agonists to boost the efficacy of immunotherapy. **F** “Cold” tumors lack activated tumor-specific T cells, which may contribute to primary resistance to ICBs. Effective combination therapy can turn these tumors into hot tumors that are sensitive to ICBs

Some of these therapies are displayed visually in Fig. 2. PD-1 inhibitors nivolumab (Opdivo), pembrolizumab (Keytruda), cemiplimab (Libtayo) and PD-L1 inhibitors atezolizumab (Tecentriq), avelumab (Bavencio) and durvalumab (Imfinzi) are the PD-(L)1 inhibitors already approved. Numerous clinical trials have been designed to study the efficacy or safety of these approved PD-(L)1 inhibitors in combination with other approved standard treatment regimens. Key information on those clinical trials that have been completed is summarized in Table 1. As can be seen from the table, among those completed clinical trials, more than half of the combination regimens were based on the anti-PD-1 antibody pembrolizumab and nivolumab. The number of combination regimens of the two anti-PD-L1 antibodies atezolizumab and durvalumab also exceeded 30. There are few strategies regarding the combination therapy of cemiplimab and avelumab. From the combination regimen of nivolumab, it can be seen that nivolumab combined with CTLA-4 antibody ipilimumab with/without other treatment strategies has the largest number. Regarding the combination regimen of pembrolizumab, it can be seen that the number of regimens with combined targeted therapy (including anti-angiogenic therapy) is the largest. The number of regimens combined with chemotherapy was the second largest. Because these combination regimens are based on approved treatments, many of them are already approved for cancer treatment.

**Table 1:** Completed clinical trials of approved PD-1/PD-L1 inhibitors in combination with other approved treatment strategy

Target	Checkpoint inhibitors	Combined intervention	Combined category	Conditions	Phase	Trial number
PD-1	Nivolumab	Tivozanib	Anti-angiogenesis therapy	RCC	Phase 1, 2	NCT03136627
		Cabozantinib	Anti-angiogenesis therapy	Breast cancer	Phase 2	NCT03316586
		Bevacizumab	Anti-angiogenesis therapy	Hepatocellular carcinoma	Phase 2	NCT04393220
		Carotuximab	Anti-angiogenesis therapy	NSCLC	Phase 1	NCT03181308
		Pomalidomide	Anti-angiogenesis therapy	Nervous system lymphoma	Phase 1	NCT03798314
		Ramucirumab	Anti-angiogenesis therapy	Gastric cancer; GEJ Cancer	Phase 1, 2	NCT02999295
		Pazopanib + sunitinib + ipilimumab	Anti-angiogenesis therapy + targeted therapy (multiple kinases) + CTLA-4 antibody	RCC	Phase 1	NCT01472081/CheckMate 016
		Pemetrexed + paclitaxel + veliparib + carboplatin	Chemotherapy	NSCLC	Phase 1	NCT02944396
		Bendamustine hydrochloride	Chemotherapy	Hodgkin's lymphoma	Phase 1, 2	NCT03343652
		Idarubicin + cytarabine + solu-medrol + dexamethasone	Chemotherapy	AML	Phase 1, 2	NCT02464657
		Carboplatin + pemetrexed + ipilimumab	Chemotherapy + CTLA-4 antibody	NSCLC	Phase 2	NCT03256136
		TG4010 + chemotherapy	Chemotherapy + Other treatment	NSCLC	Phase 2	NCT03353675
		Cyclophosphamide + fludarabine + TIL infusion + interleukin-2 + ipilimumab	Chemotherapy + other treatment + CTLA-4 antibody	Ovarian Cancer	Phase 1, 2	NCT03287674
PD-1	Nivolumab	Ipilimumab	CTLA-4 antibody	Melanoma	Phase 1	NCT01621490
		Ipilimumab	CTLA-4 antibody	Melanoma	Phase 3	NCT02599402
		Ipilimumab	CTLA-4 antibody	Melanoma	Phase 2	NCT02320058
		Ipilimumab	CTLA-4 antibody	ED-SCLC	Phase 3	NCT02538666
		Ipilimumab	CTLA-4 antibody	Melanoma	Phase 3	NCT02714218
		Ipilimumab	CTLA-4 antibody	Melanoma	Phase 3	NCT03068455
		Ipilimumab	CTLA-4 antibody	Urothelial carcinoma	Phase 1	NCT03387761
		Ipilimumab	CTLA-4 antibody	RCC	Phase 2	NCT03029780
		Ipilimumab	CTLA-4 antibody	MPM	Phase 2	NCT02716272
		Ipilimumab	CTLA-4 antibody	Melanoma	n.a	NCT03438279
		Ipilimumab	CTLA-4 antibody	Melanoma	Phase 1, 2	NCT02941744
		Ipilimumab	CTLA-4 antibody	Melanoma	Phase 2	NCT02731729
		Ipilimumab	CTLA-4 antibody	Malignant pleural mesothelioma	Phase 2	NCT03048474
		Ipilimumab	CTLA-4 antibody	Melanoma	Phase 2	NCT01783938/CheckMate 064
		Ipilimumab	CTLA-4 antibody	Cancer		NCT03165409
		Ipilimumab + SBRT	CTLA-4 antibody + radiotherapy	RCC; kidney cancer	Phase 2	NCT03065179
		Ipilimumab + radiotherapy	CTLA-4 antibody + radiotherapy	Melanoma	Phase 1	NCT02659540
		Ipilimumab + surgery	CTLA-4 antibody + surgery	Head and neck carcinoma	Phase 1, 2	NCT03003637
		Ipilimumab + enzalutamide	CTLA-4 antibody + targeted therapy (AR)	Prostate cancer	Phase 2	NCT02601014
		Interferon γ	Other treatment	Solid tumor	Phase 1	NCT02614456
		Interleukin-2	Other treatment	RCC	Phase 1, 2	NCT02989714
PD-1	Nivolumab	Tumor biopsy specimens + blood samples	Other treatment	Melanoma	n.a	NCT03348891
		JS001 + pembrolizumab	PD-1 antibody	Carcinoma; hepatocellular	Phase 2	NCT03939975
		BDCA-1 + myDC + avelumab + ipilimumab	PD-L1 antibodies + CTLA-4 antibody + Other treatment	Solid tumor	Phase 1	NCT03707808
		Mogamulizumab	Targeted therapy (CCR4)	Solid tumor	Phase 1	NCT02476123
		Mogamulizumab	Targeted therapy (CCR4)	Solid tumor	Phase 1, 2	NCT02705105
		Mogamulizumab	Targeted therapy (CCR4)	Solid cancer	Phase 1	NCT02946671
		Brentuximab vedotin	Targeted therapy (CD30)	Hodgkin lymphoma	Phase 1, 2	NCT02572167
		Brentuximab vedotin	Targeted therapy (CD30)	Hodgkin lymphoma	Phase 3	NCT03138499
		Daratumumab	Targeted therapy (CD38)	NSCLC;TNBC	Phase 1, 2	NCT03098550
		X4P-001	Targeted therapy (CXCR4)	RCC	Phase 1, 2	NCT02923531
		Cetuximab + cisplatin + radiotherapy	Targeted therapy (EGFR) + chemotherapy + radiotherapy	Squamous cell head and neck cancer	Phase 3	NCT03349710
		Entinostat	Targeted therapy (HDAC)	Cholangiocarcinoma; pancreatic adenocarcinoma	Phase 2	NCT03250273
		Vopratelimab + ipilimumab + pembrolizumab	Targeted therapy (ICOS) + CTLA-4 antibody + PD-1 antibody	Solid tumor	Phase 1, 2	NCT02904226
		Binimetinib + ipilimumab	Targeted therapy (MEK1/2) + CTLA-4 antibody	Colorectal cancer	Phase 2	NCT03271047
		Regorafenib	Targeted therapy (multiple kinases)	Solid tumor	Phase 1, 2	NCT03406871
PD-1	Nivolumab	Dasatinib	Targeted therapy (multiple kinases)	Myeloid leukemia	Phase 1	NCT02011945
		BBI503 + doxorubicin + pembrolizumab + paclitaxel + sunitinib	Targeted therapy (multiple kinases) + Chemotherapy	Cancer	Phase 1	NCT02483247
		Omaveloxolone capsules + ipilimumab	Targeted therapy (NF-κB) + CTLA-4 antibody	Melanoma	Phase 1, 2	NCT02259231
		INCAGN01949 + ipilimumab	Targeted therapy (OX40) + CTLA-4 antibody	Malignancy	Phase 1, 2	NCT03241173
		Elotuzumab + pomalidomide + dexamethasone	Targeted therapy (SLAMF7)	Myeloma	Phase 2	NCT02612779
		BBI608 + ipilimumab + pembrolizumab	Targeted therapy (STAT3) + CTLA-4 antibody + PD-1 antibody	Cancer	Phase 1	NCT02467361
		DV281 + breath actuated nebulizer	Targeted therapy (TLR) + other treatment	NSCLC	Phase 1	NCT03326752
		DS-8273a	Targeted therapy (TRAIL-R2)	Melanoma	Phase 1	NCT02983006
	Pembrolizumab	Bevacizumab	Anti-angiogenesis therapy	RCC	Phase 1, 2	NCT02348008
		Anlotinib	Anti-angiogenesis therapy	NSCLC	Phase 1, 2	NCT04670107
		Pazopanib	Anti-angiogenesis therapy	RCC	Phase 1	NCT02014636/KEYNOTE-018
		Lenalidomide	Anti-angiogenesis therapy	Blood Cancer	Phase 1	NCT01953692/KEYNOTE-013
		Bevacizumab	Anti-angiogenesis therapy	GBM	Phase 2	NCT02337491
		Bevacizumab + cyclophosphamide	Anti-angiogenesis therapy + chemotherapy	Ovarian cancer; fallopian tube cancer; peritoneal cancer	Phase 2	NCT02853318
		Doxorubicin	Chemotherapy	Endometrial cancer	Phase 2	NCT03276013
		mFOLFOX6	Chemotherapy	Colorectal cancer	Phase 2	NCT02375672
PD-1	Pembrolizumab	Nab-paclitaxel	Chemotherapy	NSCLC	Phase 1, 2	NCT02733250
		Azacitidine	Chemotherapy	Colorectal cancer	Phase 2	NCT02260440
		Nab-paclitaxel + epirubicin + cyclophosphamide	Chemotherapy	TNBC	Phase 2	NCT03289819
		Nab-paclitaxel + doxorubicin + cyclophosphamide + carboplatin + paclitaxel	Chemotherapy	TNBC	Phase 1	NCT02622074/KEYNOTE-173
		Oxaliplatin + capecitabine	Chemotherapy	BTC	Phase 2	NCT03111732
		Docetaxel	Chemotherapy	NSCLC	Phase 2	NCT02574598
		Pemetrexed + carboplatin + cisplatin	Chemotherapy	NSCLC	Phase 2	NCT03664024/KEYNOTE-782
		Carboplatin + cisplatin + etoposide	Chemotherapy	SCLC	Phase 3	NCT03066778
		Decitabine	Chemotherapy	Myeloid leukemia	Phase 1, 2	NCT02996474
		Cisplatin + 5-FU + capecitabine	Chemotherapy	Gastric cancer; gastroesophageal junction adenocarcinoma	Phase 2	NCT02335411/KEYNOTE-059
		Chemotherapy	Chemotherapy	Gastric cancer	Phase 2	NCT02918162
		Doxorubicin hydrochloride	Chemotherapy	Sarcoma	Phase 1, 2	NCT02888665
		Carboplatin	Chemotherapy	Ovarian cancer; fallopian tube cancer; peritoneal cancer	Phase 1, 2	NCT03029598
		Cyclophosphamide	Chemotherapy	MBC	Phase 2	NCT03139851
PD-1	Pembrolizumab	Paclitaxel + carboplatin + bevacizumab + pemetrexed + ipilimumab + erlotinib + gefitinib	Chemotherapy + anti-angiogenesis therapy + CTLA-4 antibody + targeted therapy (EGFR)	NSCLC	Phase 1, 2	NCT02039674/KEYNOTE-021
		Cisplatin + pemetrexed + carboplatin + paclitaxel + nab-paclitaxel + ipilimumab + etoposide + pegfilgrastim	Chemotherapy + CTLA-4 antibody + other treatment	NSCLC; SCLC	Phase 1	NCT01840579
		Niraparib + dostarlimab	Chemotherapy + PD-1 antibody	NSCLC	Phase 2	NCT03308942
		Cyclophosphamide + doxorubicin hydrochloride + prednisone + rituximab + vincristine sulfate	Chemotherapy + Targeted therapy (CD20)	Lymphoma	Phase 1	NCT02541565
		PegIFN-2b + ipilimumab	CTLA4 antibody + other treatment	RCC; Melanoma	Phase 1, 2	NCT02089685/KEYNOTE-29
		Talimogene laherparepvec	Oncolytic virus therapy	Squamous cell carcinoma of the head and neck	Phase 1	NCT02626000/KEYNOTE-137
		alfa-2b (HDI)	Other treatment	Melanoma	Phase 1	NCT02339324
		Pegzilarginase	Other treatment	SCLC	Phase 1, 2	NCT03371979
		PegIFN-2b	Other treatment	Melanoma	Phase 1	NCT02112032
		Sintilimab	PD-1 antibody	NSCLC	n.a	NCT05059951
		SABR	Radiotherapy	Breast cancer	Phase 1	NCT02303366
		Radiation therapy	Radiotherapy	Malignancy	Phase 1	NCT02987166
		Radiation therapy	Radiotherapy	RCC	Phase 2	NCT02599779
		SBRT	Radiotherapy	Urothelial cancer	Phase 1	NCT02826564
PD-1	Pembrolizumab	Intensity modulated radiation therapy	Radiotherapy	Squamous cell carcinoma of the head and neck	Phase 2	NCT03057613
		SABR	Radiotherapy	RCC	Phase 1, 2	NCT02855203
		Radiotherapy	Radiotherapy	Cancer	Phase 1	NCT02303990
		Hypofractionated stereotactic irradiation (HFSRT) + bevacizumab	Radiotherapy + anti-angiogenesis therapy	Glioma	Phase 1	NCT02313272
		Radiation therapy + cisplatin	Radiotherapy + chemotherapy	Squamous cell carcinoma	Phase 1, 2	NCT02759575
		Docetaxel + doxorubicin hydrochloride + intensity-modulated radiation therapy + therapeutic conventional surgery	Radiotherapy + chemotherapy + surgery	Thyroid cancer	Phase 2	NCT03211117
		Brachytherapy	Radiotherapy + other treatment	Esophageal cancer	Phase 1	NCT02642809
		Radiation + Vitamin D + aspirin + lansoprazole + cyclophosphamide + curcumin	Radiotherapy + other treatment + chemotherapy + targeted therapy (p300/CREB)	Cervical cancer; uterine cancer	Phase 2	NCT03192059
		Radiotherapy + rituximab + autologous dendritic cells + GM-CSF	Radiotherapy + targeted therapy (CD20) + other treatment	Follicular lymphoma	Phase 2	NCT02677155
		SBRT + trametinib	Radiotherapy + targeted therapy(MEK1/2)	Pancreatic cancer	Phase 2	NCT02704156
		Dabrafenib + trametinib	Targeted therapy (BRAF) + targeted therapy (MEK)	Melanoma	Phase 1, 2	NCT02130466/KEYNOTE-022
PD-1	Pembrolizumab	Acalabrutinib	Targeted therapy (BTK)	Ovarian cancer	Phase 2	NCT02537444/KEYNOTE191
		Acalabrutinib	Targeted therapy (BTK)	Head and neck squamous cell carcinoma	Phase 2	NCT02454179
		Acalabrutinib	Targeted therapy (BTK)	NSCLC	Phase 2	NCT02448303
		Maraviroc	Targeted therapy (CCR5)	Colorectal cancer	Phase 1	NCT03274804
		Abemaciclib + gemcitabine + ramucirumab + samotolisib	Targeted therapy (CDK) + chemotherapy + anti-angiogenesis therapy + targeted therapy (PI3K)	NSCLC	Phase 1	NCT02079636
		Necitumumab	Targeted therapy (EGFR)	NSCLC	Phase 1	NCT02451930
		Afatinib	Targeted therapy (EGFR)	Squamous cell carcinoma of the lung	Phase 2	NCT03157089
		PEGPH20	Targeted therapy (HA)	Solid tumor	Phase 1	NCT02563548
		Entinostat	Targeted therapy (HDAC)	Solid tumor	Phase 1	NCT02909452
		Vismodegib	Targeted therapy (hedgehog)	Basal cell skin cancer	Phase 1, 2	NCT02690948
		Margetuximab	Targeted therapy (HER2)	Gastric cancer; gastroesophageal junction cancer	Phase 1, 2	NCT02689284
		Eribulin mesylate	Targeted therapy (microtubule)	TNBC	Phase 1, 2	NCT02513472
		Eribulin	Targeted therapy (microtubule)	HR + /HER2- MBC	Phase 2	NCT03222856
		Anlotinib	Targeted therapy (multiple kinases)	Ovarian cancer	n.a	NCT05188781
		Axitinib	Targeted therapy (multiple kinases)	RCC	Phase 1	NCT02133742
		Lenvatinib	Targeted therapy (multiple kinases)	Gastric cancer	Phase 2	NCT03609359
		Lenvatinib	Targeted therapy (multiple kinases)	Solid tumor	Phase 1	NCT03006887
PD-1	Pembrolizumab	Saroglitazar	Targeted therapy (multiple kinases)	Biliary cancer	Phase 2	NCT02703714
		Niraparib	Targeted therapy (PARP)	TNBC; ovarian cancer	Phase 1, 2	NCT02657889
		Ibrutinib	Targeted therapy (brutons tyrosine kinase)	Non-Hodgkin lymphoma	Phase 1	NCT02950220
		Ibrutinib + everolimus + docetaxel + paclitaxel + cetuximab	Targeted therapy (brutons tyrosine kinase) + targeted therapy (mTOR) + targeted therapy (EGFR) + chemotherapy	Gastric adenocarcinoma; genitourinary adenocarcinoma	Phase 1, 2	NCT02599324
		Afatinib dimaleate	Targeted therapy (EGFR)	NSCLC	Phase 1	NCT02364609
		Platinum + afatinib	Targeted therapy (EGFR) + chemotherapy	Squamous Cell Carcinoma of the Lung		NCT04552535
		Pemigatinib + gemcitabine + cisplatin + docetaxel + trastuzumab	Targeted therapy (FGFR) + targeted therapy (HER2) + chemotherapy	Malignancies	Phase 1, 2	NCT02393248
		ImmunoPulse IL-12	Targeted therapy (IL-12)	Melanoma	Phase 2	NCT02493361
		Encorafenib + binimetinib	Targeted therapy (RAF) + targeted therapy (MEK1/2)	Melanoma	Phase 1, 2	NCT02902042
		IMP321 (eftilagimod alpha)	Targeted therapy (soluble LAG-3 protein)	Melanoma	Phase 1	NCT02676869
		Lenalidomide + dexamethasone	Targeted therapy (TNF-α)	Myeloma	Phase 2	NCT02880228
		Paricalcitol	Targeted therapy (vitamin D receptor)	Pancreatic cancer	Phase 2	NCT03331562
	Cemiplimab	Ipilimumab	CTLA-4 antibody	Lung cancer	Phase 2	NCT03430063
		Ipilimumab + chemotherapy	CTLA-4 antibody + chemotherapy	Lung cancer	Phase 3	NCT03515629
PD-1	Cemiplimab	Hypofractionated radiotherapy + cyclophosphamide + docetaxel + carboplatin + GM-CSF + paclitaxel + pemetrexed	Radiotherapy + chemotherapy	Malignancy	Phase 1	NCT02383212
		Isatuximab	Targeted therapy (CD38)	Malignancy	Phase 1, 2	NCT03367819
PD-L1	Atezolizumab	Anlotinib	Anti-angiogenesis therapy	NSCLC	Phase 1, 2	NCT04670107
		Bevacizumab + carboplatin + paclitaxel	Anti-angiogenesis therapy + chemotherapy	NSCLC	Phase 3	NCT02366143
		Bevacizumab + gemcitabine + leucovorin + Nab-paclitaxe + oxaliplatin + capecitabine + cisplatin	Anti-angiogenesis therapy + chemotherapy	Solid tumor	Phase 1	NCT02715531
		Bevacizumab + interferon alfa-2b + PEG-interferon alfa-2a + ipilimumab + obinutuzumab	Anti-angiogenesis therapy + CTLA-4 antibody + targeted therapy (CD20) + other treatment	Solid tumor	Phase 1	NCT02174172
		Bevacizumab + cobimetinib	Anti-angiogenesis therapy + targeted therapy (MEK1)	Gastrointestinal tumor	Phase 1	NCT02876224
		Bevacizumab + sunitinib	Anti-angiogenesis therapy + targeted therapy (RTK)	RCC	Phase 2	NCT01984242
		Bevacizumab + sunitinib	Anti-angiogenesis therapy + targeted therapy (RTK)	RCC	Phase 3	NCT02420821
PD-L1	Atezolizumab	5-FU + bevacizumab + carboplatin + leucovorin + nab-paclitaxel + oxaliplatin + paclitaxel + pemetrexed	Chemotherapy + anti-angiogenesis therapy	Solid tumor	Phase 1	NCT01633970
		Azacitidine	Chemotherapy	Myelodysplastic syndromes	Phase 1	NCT02508870
		Nab-Paclitaxel	Chemotherapy	TNBC	Phase 3	NCT02425891/IMpassion130
		Carboplatin + pemetrexed	Chemotherapy	NSCLC	Phase 3	NCT02367781
		Carboplatin + nab-paclitaxel + paclitaxel	Chemotherapy	NSCLC	Phase 3	NCT02367794/IMpower131
		Carboplatin + cyclophosphamide	Chemotherapy	Breast cancer; cervix cancer; ovarian cancer; endometrial cancer	Phase 1	NCT02914470
		Carboplatin + paclitaxel + bevacizumab	Chemotherapy + anti-angiogenesis therapy	Ovarian cancer	Phase 1, 2	NCT03394885
		Bendamustine + cyclophosphamide + doxorubicin + obinutuzumab + prednisone + vincristine + rituximab	Chemotherapy + targeted therapy (CD20)	DLBCL	Phase 1, 2	NCT02596971
		Carboplatin + docetaxel + pertuzumab + trastuzumab + trastuzumab emtansine + doxorubicin + cyclophosphamide	Chemotherapy + targeted therapy (HER2)	TNBC	Phase 1	NCT02605915
PD-L1	Atezolizumab	Talimogene laherparepvec	Oncolytic virus therapy	TNBC; colorectal cancer	Phase 1	NCT03256344
		Radium-223 dichloride	Radiotherapy	CRPC	Phase 1	NCT02814669
		HFRT	Radiotherapy	NSCLC	Early Phase 1	NCT02463994
		Ciforadenant	Targeted therapy (adenosine A2A receptor)	Cancer	Phase 1	NCT02655822
		Alectinib	Targeted therapy (ALK)	NSCLC	Phase 1	NCT02013219
		Obinutuzumab + polatuzumab vedotin + rituximab	Targeted therapy (CD20) + targeted therapy (CD79b)	Lymphoma	Phase 1, 2	NCT02729896
		Daratumumab	Targeted therapy (CD38)	NSCLC	Phase 1, 2	NCT03023423
		Daratumumab + lenalidomide + pomalidomide + dexamethasone	Targeted therapy (CD38) + targeted therapy (TNF-α)	Myeloma	Phase 1	NCT02431208
		Gilteritinib	Targeted therapy (FLT3/AXL)	AML	Phase 1, 2	NCT03730012
		Trastuzumab emtansine	Targeted therapy (HER2)	MBC	Phase 2	NCT02924883
		Cobimetinib	Targeted therapy (MEK1)	Solid Tumor	Phase 1	NCT01988896
		Cobimetinib + Paclitaxel + nab-paclitaxel	Targeted therapy (MEK1) + chemotherapy	TNBC	Phase 2	NCT02322814
		Cobimetinib + regorafenib	Targeted therapy (MEK1) + targeted therapy (multiple kinases)	Colorectal Cancer	Phase 3	NCT02788279
		Cobimetinib + venetoclax	Targeted therapy (MEK1) + targeted therapy (Bcl-2)	Myeloma	Phase 1, 2	NCT03312530
PD-L1	Atezolizumab	Cobimetinib + vemurafenib	Targeted therapy (MEK1) + targeted therapy (BRAF)	Melanoma	Phase 1	NCT01656642
		Rucaparib	Targeted therapy (PARP)	Gynecologic cancer; TNBC	Phase 1	NCT03101280
		Lenalidomide + obinutuzumab	Targeted therapy (TNF-α) + targeted therapy (CD20)	Follicular lymphoma	Phase 1, 2	NCT02631577
	Durvalumab	Bevacizumab	Anti-angiogenesis therapy	HER-2 negative breast cancer	Early Phase 1	NCT02802098
		Paclitaxel	Chemotherapy	TNBC	Phase 1, 2	NCT02628132
		Azacitidine	Chemotherapy	Solid tumor	Phase 2	NCT02811497
		Nab-paclitaxel + epirubicin + cyclophosphamide	Chemotherapy	TNBC	Phase 2	NCT02685059
		Tremelimumab	CTLA-4 antibody	Head and Neck cancer	Phase 3	NCT02369874
		Tremelimumab	CTLA-4 antibody	Solid tumor	Phase 1	NCT02261220
		Tremelimumab	CTLA-4 antibody	HER2 negative breast cancer	Phase 2	NCT02536794
		Tremelimumab	CTLA-4 antibody	Breast cancer; ovarian cancer; colorectal cancer; cervical cancer; RCC	Phase 1	NCT01975831
		Tremelimumab	CTLA-4 antibody	Pancreatic ductal carcinoma	Phase 2	NCT02558894
		Tremelimumab	CTLA-4 antibody	NSCLC	Phase 1	NCT02000947
		Tremelimumab	CTLA-4 antibody	Head and neck cancer	Phase 1	NCT02262741
PD-L1	Durvalumab	Tremelimumab	CTLA-4 antibody	Bladder cancer	Phase 2	NCT03430895
		Tremelimumab	CTLA-4 antibody	Malignancy	Phase 1	NCT02978482
		Tremelimumab	CTLA-4 antibody	Gastric cancer; gastroesophageal junction adenocarcinoma	Phase 1, 2	NCT02340975
		Tremelimumab	CTLA-4 antibody	Colorectal cancer	Phase 2	NCT03007407
		Tremelimumab	CTLA-4 antibody	Solid malignancy	Phase 1	NCT02141347
		Tremelimumab	CTLA-4 antibody	Prostate cancer	Phase 2	NCT03204812
		Tremelimumab + paclitaxel + carboplatin + etoposide + gemcitabine + nab-paclitaxel + 5FU + leucovorin + gemcitabine + cisplatin	CTLA-4 antibody + chemotherapy	Solid tumor	Phase 1	NCT02658214
		Tremelimumab + SBRT	CTLA-4 antibody + radiotherapy	Pancreatic cancer	Phase 1, 2	NCT02311361
		Mogamulizumab + tremelimumab	CTLA-4 antibody + targeted therapy (CCR4)	Solid tumor	Phase 1	NCT02301130
		Tremelimumab + cetuximab + 5FU	CTLA-4 antibody + targeted therapy (EGFR) + chemotherapy	Head and Neck cancer	Phase 3	NCT02551159
		Tremelimumab + AZD9150	CTLA-4 antibody + targeted therapy (STAT3)	DLBCL	Phase 1	NCT02549651
		HFRT + SBRT + tremelimumab	Radiotherapy + CTLA-4 antibody	SCLC	Phase 2	NCT02701400
PD-L1	Durvalumab	Brain radiotherapy + stereotactic radiosurgery + tremelimumab + HER2 directed therapy	Radiotherapy + CTLA-4 antibody + other treatment	Breast cancer	n.a	NCT02563925
		Ablation + radiotherapy + tremelimumab	Surgery + radiotherapy + CTLA-4 antibody	Colorectal cancer	Phase 2	NCT03122509
		Ensartinib	Targeted therapy (ALK)	NSCLC	Phase 1, 2	NCT02898116
		Dabrafenib + trametinib	Targeted therapy (BRAF) + targeted therapy (MEK1/2)	Melanoma	Phase 1	NCT02027961
		Ibrutinib	Targeted therapy (brutons tyrosine kinase)	Solid tumor	Phase 1, 2	NCT02403271
		Ibrutinib	Targeted therapy (brutons tyrosine kinase)	DLBCL; follicular lymphoma	Phase 1, 2	NCT02401048
		Daratumumab	Targeted therapy (CD38)	Myeloma	Phase 2	NCT03000452
		Pexidartinib	Targeted therapy (CSF1R)	Pancreatic cancer; colorectal cancer	Phase 1	NCT02777710
		AZD5069 + nab-paclitaxel + gemcitabine	Targeted therapy (CXCR2) + chemotherapy	Pancreatic ductal adenocarcinoma	Phase 1, 2	NCT02583477
		Gefitinib	Targeted therapy (EGFR)	NSCLC	Phase 1	NCT02088112
		Selumetinib + tremelimumab	Targeted therapy (MEK) + CTLA-4 antibody	Solid tumor	Phase 1	NCT02586987
		Eribulin	Targeted therapy (microtubule)	Breast cancer; ovarian cancer	Phase 1	NCT03430518
		Olaparib	Targeted therapy (PARP)	Squamous cell carcinoma of the head and neck	Phase 2	NCT02882308
PD-L1	Durvalumab	Olaparib	Targeted therapy (PARP)	Bladder cancer	Phase 2	NCT03534492
		Olaparib + bevacizumab	Targeted therapy (PARP) + anti-angiogenesis therapy	Solid tumor	Phase 1, 2	NCT02734004
	Avelumab	Axitinib	Anti-angiogenesis therapy	GBM	Phase 2	NCT03291314
		Axitinib	Anti-angiogenesis therapy	RCC	Phase 1	NCT02493751
		Axitinib	Anti-angiogenesis therapy	HCC	Phase 1	NCT03289533
		Talazoparib + chemotherapy	Chemotherapy + targeted therapy (PARP)	Ovarian cancer	Phase 3	NCT03642132
		HFRT	Radiotherapy	GMB	Phase 2	NCT02968940
		SAR	Radiotherapy	NSCLC	Early Phase 1	NCT03158883
		Cisplatin + 5-FU + mitomycin + radiation therapy	Radiotherapy + chemotherapy	Bladder cancer	Phase 2	NCT03617913
		Radiotherapy + cetuximab	Radiotherapy + chemotherapy + targeted therapy (EGFR)	Squamous cell carcinoma of the head and neck	Phase 1	NCT02938273
		Magrolimab	Targeted therapy (CD47)	Ovarian cancer	Phase 1	NCT03558139
		Cetuximab + gemcitabine + cisplatin + carboplatin	Targeted therapy (EGFR) + chemotherapy	NSCLC	Phase 2	NCT03717155

*n.a. *not applicable, *SABR* stereotactic ablative body radiosurgery, *HFSRT *hypofractionated stereotactic irradiation, *SAR *stereotactic ablative radiotherapy, *RCC* renal cell carcinoma, *NSCLC *non-small-cell lung cancer, *TNBC* triple-negative breast cancer, *SCLC *small-cell lung cancer, *ED-SCLC* extensive-stage disease small-cell lung cancer, *MPM *malignant pleural mesothelioma, *AML *acute myeloid leukemia, *MBC *metastatic breast cancer, *BTC *biliary tract carcinoma, *GBM* glioblastoma multiforme, *CRPC* castrate-resistant prostate cancer, *DLBCL* diffuse large B-cell lymphoma, *GEJ Adenocarcinoma* gastroesophageal junction adenocarcinoma, *HCC* hepatocellular carcinoma

In addition to the above combination experiments, there are a large number of clinical trials studying the efficacy and side effects of approved/investigational PD-(L)1 inhibitors in combination with other cancer treatment regimens. Key information on representative ongoing clinical trials of such combination therapy is shown in Table 2. As can be seen from the table, most of the combination strategies are PD-1/PD-L1 blockade combined with chemotherapy, targeted therapy, radiotherapy and other immune checkpoint inhibitors. Chemotherapy, targeted therapy and radiotherapy are classic strategies for cancer treatment, and most approved cancer treatment strategies belong to them. Therefore, combination regimen containing these treatment strategies is likely to show numerous breakthroughs. PD-1/PD-L1 blockade has also been combined with many biotherapy regimens, such as cell therapy and vaccine. In addition, PD-1/PD-L1 blockade has also been combined with many novel cancer treatment options, such as electric field therapy, which has shown excellent efficacy in the treatment of glioma. More than half of the combination regimens belong to dual combination therapy, such as PD-1/PD-L1 blockade combined with chemotherapy. We can also see clinical trials with triple combination therapy. The results of these clinical trials will bring valuable data to improve the efficacy of PD-1/PD-L1 blockade.

**Table 2:** Representative ongoing clinical trials for PD-1/PD-L1 blockade in combination with other cancer treatment regimens

Interventions	Target	Conditions	Phase	Development status	Trial number
*1. PD-1/PD-L1 blockade + Other type of immune checkpoint inhibitors*					
Approved immune-checkpoint inhibitors therapies	PD-1 + PD-L1 + CTLA-4	Melanoma; NSCLC	Phase 4	Recruiting	NCT03673332
Nivolumab + ipilimumab	PD-1 + CTLA-4	NSCLC; SCLC	Phase 2	Recruiting	NCT03823625, NCT03285321, NCT03333616
Nivolumab + ipilimumab	PD-1 + CTLA-4	NSCLC	Phase 2	Active, not recruiting	NCT03001882, NCT03091491, NCT03262779
Nivolumab + ipilimumab	PD-1 + CTLA-4	NSCLC; SCLC	Phase 2	Enrolling by invitation	NCT03083691
Nivolumab + ipilimumab	PD-1 + CTLA-4	NSCLC; SCLC	Phase 3	Active, not recruiting	NCT02538666
Nivolumab + ipilimumab	PD-1 + CTLA-4	NSCLC; SCLC	Phase 4	Active, not recruiting	NCT02869789
Pembrolizumab + ipilimumab	PD-1 + CTLA-4	NSCLC	Phase 3	Active, not recruiting	NCT03302234
Nivolumab + relatlimab	PD-1 + LAG-3	NSCLC	Phase 2	Recruiting	NCT04205552, NCT04623775
BI 754091 + BI 754111	PD-1 + LAG-3	NSCLC	Phase 1	Active, not recruiting	NCT03156114
XmAb®23104 + ipilimumab	PD-1/ICOS + CTLA-4	Melanoma; NSCLC; SCLC	Phase 1	Recruiting	NCT03752398
Nivolumab + ipilimumab or chemotherapy	PD-1 + CTLA-4/chemotherapy	NSCLC; SCLC	Phase 2	Recruiting	NCT03158129
Nivolumab + ipilimumab or nivolumab + platinum-doublet chemotherapy	PD-1 + CTLA-4 / PD-1 + chemotherapy	NSCLC	Phase 3	Recruiting	NCT02477826
Durvalumab + tremelimumab + chemotherapy	PD-L1 + CTLA-4 + chemotherapy	NSCLC	Phase 3	Recruiting	NCT03164616
*2. PD-1/PD-L1 blockade + Chemotherapy*					
Nivolumab + decitabine + tetrahydrouridine	PD-1 + chemotherapy	NSCLC; SCLC	Phase 2	Active, not recruiting	NCT02664181
Nivolumab + gemcitabine	PD-1 + chemotherapy	NSCLC	Phase 4	Not yet recruiting	NCT04331626
Nivolumab + docetaxel	PD-1 + chemotherapy	NSCLC	Phase 3	Recruiting	NCT03906071
Nivolumab/pembrolizumab + chemotherapy	PD-1 + chemotherapy	SCLC	Not Applicable	Not yet recruiting	NCT04306042
Nivolumab + carboplatin + paclitaxel	PD-1 + chemotherapy	NSCLC	Phase 2	Recruiting	NCT02259621
Nivolumab + platinum-based chemotherapy	PD-1 + chemotherapy	NSCLC	Phase 2	Recruiting	NCT03823625
Nivolumab + paclitaxel + carboplatin AUC5	PD-1 + chemotherapy	NSCLC	Phase 1	Recruiting	NCT04699721
Nivolumab + carboplatin + nab-paclitaxel	PD-1 + chemotherapy	NSCLC	Phase 2	Recruiting	NCT04015778
Nivolumab + temozolomide	PD-1 + chemotherapy	SCLC	Phase 2	Active, not recruiting	NCT03728361
Nivolumab + decitabine + tetrahydrouridine	PD-1 + chemotherapy	NSCLC; SCLC	Phase 2	Active, not recruiting	NCT02664181
Nivolumab + irinotecan	PD-1 + chemotherapy	SCLC	Phase 1	Recruiting	NCT04173325
Nivolumab + carboplatin + cisplatin + etoposide	PD-1 + chemotherapy	SCLC	Phase 2	Active, not recruiting	NCT03382561
Toripalimab + platinum-based chemotherapy	PD-1 + chemotherapy	NSCLC	Phase 2	Recruiting	NCT05055583
Opdivo/Keytruda + tirapazamine	PD-1 + chemotherapy	NSCLC; SCLC	Phase 2	Recruiting	NCT03259867
Camrelizumab + nab-paclitaxel	PD-1 + chemotherapy	NSCLC	Phase 2	Recruiting	NCT04167774
PD-1 inhibitor + chemotherapy	PD-1 + chemotherapy	NSCLC	Phase 2	Recruiting	NCT04941417
Pembrolizumab + pemetrexed + gemcitabine + cisplatin + carboplatin	PD-1 + chemotherapy	NSCLC	Phase 2	Recruiting	NCT04586465
Pembrolizumab + tirapazamine	PD-1 + chemotherapy	NSCLC; SCLC	Phase 2	Recruiting	NCT04701476
Atezolizumab + gemcitabine	PD-L1 + chemotherapy	NSCLC	Phase 2	Recruiting	NCT04480372
Nivolumab + relatlimab + carboplatin + cisplatin + paclitaxel + nab-paclitaxel + pemetrexed	PD-1 + LAG-3 + chemotherapy	NSCLC	Phase 2	Recruiting	NCT04623775
Nivolumab + BMS-986012 + carboplatin + etoposide	PD-1 + fucosyl-GM1 + chemotherapy	SCLC	Phase 2	Recruiting	NCT04702880
Nivolumab + ipilimumab + platinum-doublet chemotherapy	PD-1 + CTLA-4 + chemotherapy	NSCLC	Phase 2	Active, not recruiting	NCT02659059
Nivolumab + ipilimumab + oxaliplatin	PD-1 + CTLA-4 + chemotherapy	NSCLC	Phase 1, Phase 2	Recruiting	NCT04043195
Nivolumab + ipilimumab + paclitaxel	PD-1 + CTLA-4 + chemotherapy	NSCLC	Phase 2	Active, not recruiting	NCT03573947
Nivolumab + ipilimumab + guadecitabine	PD-1 + CTLA-4 + chemotherapy	Melanoma; NSCLC	Phase 2	Not yet recruiting	NCT04250246
Nivolumab + ipilimumab + platinum-based chemotherapy	PD-1 + CTLA-4 + chemotherapy	SCLC	Phase 2	Active, not recruiting	NCT03670056
Anti-PD-1/PD-L1 monoclonal antibody + Chemotherapy + bronchoscopy-assisted interventional therapy	PD-1/PD-L1 + chemotherapy + interventional therapy	NSCLC	Phase 2, Phase 3	Not yet recruiting	NCT04702009
Immune checkpoint inhibitor + chemotherapy	PD-1/PD-L1/CTLA-4 + chemotherapy	NSCLC	Not applicable	Recruiting	NCT04807114
Durvalumab + bevacizumab + pemetrexed + cisplatin/carboplatin + SBRT	PD-L1 + EGFR + chemotherapy + SBRT	NSCLC; SCLC	Phase 2	Not yet recruiting	NCT04517526
Camrelizumab + apatinib + albumin paclitacxel	PD-1 + VEGF + chemotherapy	NSCLC; SCLC	Phase 2	Not yet recruiting	NCT04459078
Nivolumab + ipilimumab/nivolumab + chemotherapy	PD-1 + CTLA-4 / PD-1 + chemotherapy	NSCLC	Phase 3	Active, not recruiting	NCT02998528
Pembrolizumab + carboplatin-paclitaxel/nab-paclitaxel	PD-1 + chemotherapy	NSCLC	Phase 3	Active, not recruiting	NCT02775435
Atezolizumab + chemotherapy	PD-L1 + chemotherapy	NSCLC	Phase 3	Active, not recruiting	NCT02486718
Pembrolizumab + chemotherapy + radiotherapy	PD-1 + chemotherapy + radiotherapy	NSCLC	Phase 2	Active, not recruiting	NCT03631784
Atezolizumab + carboplatin/cisplatin + pemetrexed	PD-L1 + chemotherapy	NSCLC	Phase 3	Active, not recruiting	NCT02657434
*3. PD-1/PD-L1 blockade + Radiotherapy*					
Nivolumab/pembrolizumab + radiotherapy	PD-1 + radiotherapy	Melanoma	Phase 2	Recruiting	NCT04017897
Sintilimab + radiotherapy	PD-1 + radiotherapy	NSCLC; SCLC	Phase 2	Recruiting	NCT04513301
Nivolumab + radiosurgery	PD-1 + radiosurgery	NSCLC	Phase 2	Active, not recruiting	NCT02978404
Nivolumab + intensity modulated radiotherapy (IMRT)	PD-1 + IMRT	NSCLC	Phase 2	Recruiting	NCT04577638
Nivolumab + SBRT	PD-1 + SBRT	NSCLC	Phase 2	Recruiting	NCT04271384
Nivolumab + ipilimumab + radiation therapy	PD-1 + CTLA-4 + radiotherapy	NSCLC	Phase 1, Phase 2	Recruiting	NCT03168464, NCT04013542, NCT02696993
Nivolumab + ipilimumab + thoracic radiation therapy(TRT)	PD-1 + CTLA-4 + TRT	SCLC	Phase 1, Phase 2	Active, not recruiting	NCT03043599
Nivolumab + BMS-986218 + SBRT	PD-1 + CTLA-4 + SBRT	NSCLC; SCLC	Phase 1, Phase 2	Recruiting	NCT04785287
Nivolumab + ipilimumab + SBRT	PD-1 + CTLA-4 + SBRT	SCLC	Phase 1	Recruiting	NCT03223155
Durvalumab + radiotherapy	PD-L1 + radiotherapy	NSCLC	Phase 2	Recruiting	NCT04062708
Durvalumab + tremelimumab + radiation therapy	PD-L1 + CTLA-4 + radiotherapy	NSCLC	Phase 2	Active, not recruiting	NCT02888743
Atezolizumab + SBRT	PD-L1 + SBRT	NSCLC	Phase 2	Recruiting	NCT02992912
Nivolumab/pembrolizumab/atezolizumab + stereotactic body radiotherapy (SBRT)	PD-1/PD-L1 + SBRT	Melanoma; NSCLC	Phase 2	Active, not recruiting	NCT03511391
Immunotherapeutic agent + radiation	PD-1/PD-L1/CTLA-4 + radiotherapy	NSCLC	Not applicable	Active, not recruiting	NCT03035890
Immunotherapy + radiation	PD-1/PD-L1/CTLA-4 + radiotherapy	NSCLC; SCLC	Not applicable	Recruiting	NCT03705806
Nivolumab, pembrolizumab, ipilimumab or atezolizumab + SBRT	PD-1/PD-L1/CTLA-4 + SBRT	Melanoma; NSCLC; SCLC	Phase 2	Recruiting	NCT03693014
Durvalumab + bevacizumab + pemetrexed + cisplatin/carboplatin + SBRT	PD-L1 + EGFR + Chemotherapy + SBRT	NSCLC; SCLC	Phase 2	Not yet recruiting	NCT04517526
*4. PD-1/PD-L1 blockade + targeted therapy*					
Spartalizumab + PBF-509	PD-1 + AR	NSCLC	Phase 1, Phase 2	Active, not recruiting	NCT02403193
Pembrolizumab + INCB001158	PD-1 + Arg	NSCLC; SCLC	Phase 1, Phase 2	Active, not recruiting	NCT02903914
PD-1 inhibitor + metformin	PD-1 + AMPK	SCLC	Phase 2	Recruiting	NCT03994744
Nivolumab + metformin hydrochloride	PD-1 + AMPK	NSCLC	Phase 2	Active, not recruiting	NCT03048500
Nivolumab + ceritinib	PD-1 + ALK	NSCLC	Phase 1	Active, not recruiting	NCT02393625
PD-1 inhibitor + CAB-AXL-ADC	PD-1 + AXL	NSCLC	Phase 2	Recruiting	NCT04681131
PD-1 inhibitor + CAB-AXL-ADC	PD-1 + AXL	Melanoma; NSCLC	Phase 1, Phase 2	Recruiting	NCT03425279
Nivolumab + BMS-986340	PD-1 + CCR8	NSCLC; SCLC	Phase 1, Phase 2	Recruiting	NCT04895709
Spartalizumab + NIR178	PD-1 + CD73	NSCLC	Phase 2	Recruiting	NCT03207867
Nivolumab + pembrolizumab + OR2805	PD-1 + CD163	Melanoma; NSCLC; SCLC	Phase 1, Phase 2	Recruiting	NCT05094804
Nivolumab + cabiralizumab + APX005M	PD-1 + CSF1R + CD40	Melanoma; NSCLC	Phase 1	Recruiting	NCT03502330
Nivolumab + nimotuzumab	PD-1 + EGFR	NSCLC	Phase 1, Phase 2	Recruiting	NCT02947386
Nivolumab + nintedanib	PD-1 + EGFR	NSCLC; SCLC	Phase 1, Phase 2	Recruiting	NCT04046614
Nivolumab + BT5528	PD-1 + EphA2	NSCLC	Phase 1, Phase 2	Recruiting	NCT04180371
Sintilimab + pemigatinib	PD-1 + FGFR	NSCLC	Phase 2	Not yet recruiting	NCT05004974
Nivolumab + BMS-986012 + carboplatin + etoposide	PD-1 + fucosyl-GM1 + chemotherapy	SCLC	Phase 2	Recruiting	NCT04702880
Nivolumab + BMS-986012	PD-1 + fucosyl-GM1	SCLC	Phase 1, Phase 2	Active, not recruiting	NCT02247349
Nivolumab + plinabulin	PD-1 + GEF-H1	NSCLC	Phase 1	Recruiting	NCT02812667
Nivolumab + ACY-241	PD-1 + HDAC6	NSCLC	Phase 1	Active, not recruiting	NCT02635061
Nivolumab + HBI-8000	PD-1 + HDAC	Melanoma; NSCLC	Phase 1, Phase 2	Active, not recruiting	NCT02718066
PDR001 + DKY709	PD-1 + helios (IKZF2)	Melanoma; NSCLC	Phase 1	Recruiting	NCT03891953
Nivolumab + Ipilimumab + BMS-986205	PD-1 + IDO1	Melanoma; NSCLC	Phase 1, Phase 2	Active, not recruiting	NCT02658890
Nivolumab + LT-803	PD-1 + IL-15	NSCLC	Phase 1, Phase 2	Active, not recruiting	NCT02523469
Spartalizumab + JDQ443 + TNO155	PD-1 + KRAS G12C + SHP2	NSCLC	Phase 1, Phase 2	Recruiting	NCT04699188
Spartalizumab + capmatinib	PD-1 + MET	NSCLC	Phase 2	Active, not recruiting	NCT04323436
Nivolumab + glesatinib + sitravatinib + mocetinostat	PD-1 + MET/SMO + RTK + HDAC (Class I/IV)	NSCLC	Phase 2	Active, not recruiting	NCT02954991
Nivolumab + rucaparib	PD-1 + PARP	SCLC	Phase 2	Recruiting	NCT03958045
Nivolumab + copanlisib	PD-1 + PI3K	NSCLC	Phase 1	Active, not recruiting	NCT03735628
Nivolumab + eganelisib	PD-1 + PI3K-γ	Melanoma; NSCLC	Phase 1	Active, not recruiting	NCT02637531
Nivolumab + TPST-1120	PD-1 + PPARa	NSCLC; SCLC	Phase 1	Recruiting	NCT03829436
Nivolumab + COM701	PD-1 + PVRIG	NSCLC; SCLC	Phase 1	Recruiting	NCT03667716
Nivolumab + denosumab	PD-1 + RANKL	NSCLC	Phase 2	Recruiting	NCT03669523
PD-1 inhibitor + CAB-ROR2-ADC	PD-1 + ROR2	Melanoma; NSCLC	Phase 1, Phase 2	Recruiting	NCT03504488
Nivolumab + sitravatinib	PD-1 + RTKs	NSCLC	Phase 3	Recruiting	NCT03906071
Spartalizumab + TNO155 + ribociclib	PD-1 + SHP2 + CDK4/6	NSCLC	Phase 1	Recruiting	NCT04000529
PD-1 inhibitor + JAB-3068	PD-1 + SHP2	NSCLC	Phase 1, Phase 2	Recruiting	NCT04721223
Camrelizumab + famitinib	PD-1 + TKI	NSCLC	Phase 3	Recruiting	NCT05106335
Immune checkpoint inhibitor + anti-angiogenesis agents	PD-1 + VEGF	NSCLC	Not applicable	Recruiting	NCT04137588
Ezabenlimab + BI 836880	PD-1 + VEGF/Ang2	NSCLC	Phase 1	Recruiting	NCT03468426
Camrelizumab + apatinib + albumin paclitacxel	PD-1 + VEGF + chemotherapy	NSCLC; SCLC	Phase 2	Not yet recruiting	NCT04459078
Tislelizumab + anlotinib + irinotecan	PD-1 + VEGFR	SCLC	Not applicable		NCT05027100
PD-1 inhibitor + anlotinib	PD-1 + VEGFR	NSCLC; SCLC	Phase 2	Recruiting	NCT04790409
Nivolumab + anlotinib	PD-1 + VEGFR	NSCLC	Phase 2	Not yet recruiting	NCT04211896
Nivolumab + ramucirumab	PD-1 + VEGFR2	NSCLC	Phase 2	Recruiting	NCT03527108
Nivolumab + anlotinib	PD-1 + VEGFR2	NSCLC	Phase 1, Phase 2	Recruiting	NCT04507906
Nivolumab + AL3818	PD-1 + VEGFR2	NSCLC; SCLC	Phase 1, Phase 2	Recruiting	NCT04165330
Nivolumab + X-82	PD-1 + VEGFR/PDGFR	NSCLC; SCLC	Phase 1, Phase 2	Recruiting	NCT03583086
Nivolumab + ipilimumab + nintedanib	PD-1 + CTLA-4 + EGFR	NSCLC; SCLC	Phase 1, Phase 2	Active, not recruiting	NCT03377023
Nivolumab + ipilimumab + plinabulin	PD-1 + CTLA-4 + GEF-H1	NSCLC; SCLC	Phase 1, Phase 2	Recruiting	NCT03575793
Nivolumab + ipilimumab + NKTR-214	PD-1 + CTLA-4 + IL-2	Melanoma; NSCLC	Phase 1, Phase 2	Active, not recruiting	NCT02983045
Nivolumab + ipilimumab + Tocilizumab	PD-1 + CTLA-4 + IL-6	NSCLC; SCLC	Phase 2	Recruiting	NCT04940299
Nivolumab + ipilimumab + denosumab	PD-1 + CTLA-4 + RANKL	Melanoma	Phase 1, Phase 2	Recruiting	NCT03161756
Nivolumab + ipilimumab + BMS-986207	PD-1 + CTLA-4 + TIGIT	NSCLC	Phase 2	Not yet recruiting	NCT05005273
Nivolumab + ipilimumab + certolizumab/infliximab	PD-1 + CTLA-4 + TNF-a	Melanoma	Not applicable	Active, not recruiting	NCT03293784
Pembrolizumab + GEN1046	PD-1 + PD-L1 + 4-1BB	NSCLC	Phase 2	Recruiting	NCT05117242
Durvalumab/avelumab/atezolizumab/nivolumab/pembrolizumab + N-803 + PD-L1 t-haNK	PD-1/PD-L1 + IL-15 + cell therapy	Melanoma; NSCLC; SCLC	Phase 2	Active, not recruiting	NCT03228667
ICB + MDNA11	PD-1/PD-L1/CTLA-4 + IL-2	Melanoma; NSCLC	Phase 1, Phase 2	Recruiting	NCT05086692
Durvalumab + oleclumab/ceralasertib	PD-L1 + CD73/ATR	NSCLC	Phase 2	Recruiting	NCT03833440
Durvalumab + bevacizumab + pemetrexed + cisplatin/carboplatin + SBRT	PD-L1 + EGFR + chemotherapy + SBRT	NSCLC; SCLC	Phase 2	Not yet recruiting	NCT04517526
Atezolizumab + tocilizumab	PD-L1 + IL-6R	NSCLC; SCLC	Phase 1, Phase 2	Not yet recruiting	NCT04691817
Atezolizumab + cabozantinib	PD-L1 + TKI	NSCLC	Phase 1, Phase 2	Recruiting	NCT03170960
Atezolizumab + ramucirumab	PD-L1 + VEGFR-2	NSCLC	Phase 2	Active, not recruiting	NCT03689855
Durvalumab-platinum–etoposide + anlotinib	PD-L1 + VEGF	SCLC	Phase 2	Not yet recruiting	NCT04660097
Ipilimumab + osimertinib	CTLA-4 + EGFR	NSCLC	Phase 1	Recruiting	NCT04141644
*5. PD-1/PD-L1 blockade + cell therapy*					
Nivolumab + pembrolizumab + atezolizumab + FT500	PD-1 + PD-L1 + NK cell cancer immunotherapy	Melanoma; SCLC	Phase 1	Recruiting	NCT03841110
Sintilimab + CIK cell + pemetrexed + albumin paclitaxel + carboplatin	PD-1 + CIK cell therapy + chemotherapy	NSCLC	Phase 2	Not yet recruiting	NCT04836728
Nivolumab + MILs™—NSCLC + Tadalafil	PD-1 + cell therapy + PDE-5	NSCLC; SCLC	Phase 2	Active, not recruiting	NCT04069936
Nivolumab, ipilimumab, pembrolizumab, lifileucel, LN-145, LN-145-S1	PD-1 + CTLA-4 + TIL cell therapy	NSCLC	Phase 2	Recruiting	NCT03645928
Atezolizumab + cyclophosphamide + fludarabine + MAGE-A1-specific T cell receptor-transduced autologous T cells	PD-L1 + cell therapy + Chemotherapy	NSCLC; SCLC	Phase 1, Phase 2	Recruiting	NCT04639245
Nivolumab + IRX 2	PD-1 + cell therapy	Melanoma; NSCLC; SCLC	Phase 1	Active, not recruiting	NCT03758781
*6. PD-1/PD-L1 blockade + vaccine*					
ICB + ChAdOx1-MAGEA3-NYESO/MVA-MAGEA3 + chemotherapy	PD-1/PD-L1/CTLA-4 + vaccine + chemotherapy	NSCLC	Phase 1, Phase 2	Recruiting	NCT04908111
Nivolumab + ipilimumab + dendritic cell-based p53 Vaccine	PD-1 + CTLA-4 + vaccine	NSCLC; SCLC	Phase 2	Active, not recruiting	NCT03406715
Nivolumab + UCPVax	PD-1 + vaccine	NSCLC	Phase 2	Recruiting	NCT04263051
Nivolumab + pembrolizumab + pemetrexed + viagenpumatucel-L	PD-1 + DHFR + vaccine	NSCLC	Phase 1, Phase 2	Active, not recruiting	NCT02439450
Nivolumab + TG4010 + chemotherapy	PD-1 + vaccine + chemotherapy	NSCLC	Phase 2	Active, not recruiting	NCT03353675
Nivolumab + ipilimumab + GRT-C901/GRT-R902	PD-1 + CTLA-4 + vaccine	NSCLC	Phase 1, Phase 2	Active, not recruiting	NCT03639714
Nivolumab + ipilimumab + GRT-C903/GRT-R904	PD-1 + CTLA-4 + vaccine	NSCLC	Phase 1, Phase 2	Recruiting	NCT03953235
Nivolumab + pembrolizumab + recombinant human EGF-rP64K/montanide ISA 51 vaccine	PD-1 + vaccine	NSCLC	Phase 1, Phase 2	Active, not recruiting	NCT02955290
Nivolumab + ipilimumab + UV1 vaccine + leukine	PD-1 + CTLA-4 + vaccine	NSCLC; SCLC	Phase 2	Recruiting	NCT04300244
Nivolumab + pembrolizumab + GEN-009 adjuvanted vaccine	PD-1 + vaccine	NSCLC	Phase 1, Phase 2	Active, not recruiting	NCT03633110
*7. PD-1/PD-L1 blockade + Other treatment regimens*					
ICI + fecal microbial transplantation (FMT)	PD-1/PD-L1/CTLA-4 + FMT	Melanoma; NSCLC	Phase 2	Not yet recruiting	NCT04951583
Nivolumab + FMT by capsules	PD-1 + FMT	Melanoma; NSCLC	Phase 1, Phase 2	Recruiting	NCT04521075
Opdivo + Yervoy + Novocure Optune	PD-1 + CTLA-4 + electric field therapy	Melanoma	Phase 1	Not yet recruiting	NCT05004025
Camrelizumab + microwave ablation (MWA)	PD-1 + MWA	NSCLC; SCLC	Phase 2	Recruiting	NCT05053802
Pembrolizumab + laser interstitial thermotherapy (LITT)	PD-1 + LITT	Melanoma; NSCLC; SCLC	Phase 1	Recruiting	NCT04187872
Pembrolizumab + Radium-223 dichloride (Xofigo, BAY 88–8223)	PD-1 + radiotherapeutic drug	NSCLC	Phase 1	Active, not recruiting	NCT03996473
ICB + CAN-2409 + valacyclovir	PD-1/PD-L1/CTLA-4 + gene therapy	NSCLC	Phase 2	Recruiting	NCT04495153
ICB + Different sleep conditions	ICB + sleep disturbances	NSCLC; SCLC	Not Applicable	Recruiting	NCT04070651
Exercise intervention + standard oncological treatments	ICB + chemotherapy + exercise intervention	NSCLC; SCLC	Not Applicable	Recruiting	NCT04263467

Phase 4: Only after the drug is approved can it enter the phase 4 of the trial. At this stage, new uses or new populations of drugs, long-term effects and subjects' responses to different doses can be further studied

*NSCLC* non-small-cell lung cancer, *SCLC* small-cell lung cancer, *PD-1 *programmed cell death-1, *PD-L1 *PD-1 ligand, *CTLA-4 *cytotoxic T-lymphocyte-associated protein 4, *LAG-3 *lymphocyte-activation gene 3, *ICOS *inducible co-stimulator, *EGFR *epidermal growth factor receptor, *VEGF *vascular endothelial growth factor, *VEGFR *vascular endothelial growth factor receptor, *IMRT *intensity modulated radiotherapy, *SBRT *stereotactic body radiation therapy, *TRT *thoracic radiation therapy, *AR* adenosine receptor, *Arg* arginase, *AMPK *adenosine 5'-monophosphate-activated protein kinase, *ALK* anaplastic lymphoma kinase, *ATR *ATR serine/threonine kinase, *AXL *AXL receptor tyrosine kinase, *CCR8 *CC chemokine receptor 8, *CD73 *ecto-5'-nucleotidase, *CSF1R *colony stimulating factor 1 receptor, *EphA2 *ephrin A receptor 2, *FGFR *fibroblast growth factor receptor, *GEF-H1 *guanine nucleotide exchange factor H1, *HDAC *histone deacetylase, *Helios* IKAROS-family genes, *IDO1* indoleamine 2,3-dioxygenase 1, *IL-2* interleukin-2, *IL-6* interleukin-6, *IL-6R* interleukin-6 receptor, *IL-15* interleukin-15, *KRAS* KRAS proto-oncogene, *SHP2* Src homology 2 domain tyrosine phosphatases, *MET* MET proto-oncogene, *RTKs* AXL, MER, VEGFR2, PDGFR, KIT, RET, MET, DDR2, TRKA, *PARP* poly ADP ribose polymerase, *PI3K* phosphatidylinositol 3-kinase, *PPARa* peroxisome proliferator activated receptor a, *PVRIG* CD112 receptor, *RANKL* receptor activator of nuclear factor kappa-B ligand, *ROR2 *recombinant receptor tyrosine kinase like orphan receptor 2, *CDK4/6* cyclin-dependent kinase 4/6, *TKI* tyrosine kinase inhibitor, *PDGFR* platelet-derived growth factor receptor, *TIGIT* T cell immunoreceptor with Ig and ITIM domains, *TNF-a *tumor necrosis factor-a, *CIK* cytokine-induced killer, *TIL* tumor infiltrating lymphocytes, *DHFR* dihydrofolate reductase, *FMT *fecal microbial transplantation, *MWA* microwave ablation, *LITT* laser interstitial thermotherapy

### Combination therapy with PD-1 and CTLA-4 blockers

Upregulation of other immune checkpoints is a potential cause of resistance to PD-1 inhibitors. Therefore, combination therapy with other ICIs can be a crucial strategy, including the combination of PD-1 inhibitors and CTLA-4 inhibitors, which is commonly used in clinical applications.

The combination of the anti-PD-1 antibody nivolumab and the anti-CTLA-4 antibody ipilimumab was used for the first time in humans in December 2009 and targeted two unrelated pathways [130]. CTLA-4 produces a strong inhibitory signal to terminate the proliferation and activation of T cells, which can be blocked by CTLA-4 inhibitors, thus restoring the activation of T cells. Therefore, CTLA-4 mainly acts on interactive signal transmission between lymphocytes. PD-1 blocks the activation of the immune response [131]. Flow cytometry also indicated that CTLA-4 and PD-1 inhibitors target proteins in different pathways [132]. Additionally, a preclinical study demonstrated the synergistic effect of nivolumab and ipilimumab in a mouse cancer model [133].

Combination therapy was initially designed for populations that do not express PD-L1. The clinical trial CheckMate 227 (NCT02477826) reported that the efficacy of first-line combination therapy with nivolumab and ipilimumab was superior to that of platinum-doublet chemotherapy in advanced NSCLC patients with a high tumor mutation burden (TMB ≥ 10 mut/Mb); combination therapy with nivolumab and ipilimumab significantly enhanced the overall response rate (ORR, 45.3% vs. 26.9%) and median progression-free survival (mPFS, 7.2 months vs. 5.5 months) [134]. The results of the CheckMate-227 trial on advanced NSCLC immunotherapy showed that treatment with nivolumab plus ipilimumab for 4 years provided robust and long-term OS benefits for patients with advanced NSCLC compared to that for chemotherapy regardless of PD-L1 expression or histological type. However, the incidence of immune-related AEs (irAEs) is significantly higher in combination therapy than in monotherapy and requires further analyzed [135].

The NEOSTAR phase II randomized clinical trial (NCT03158129) found that the combination of nivolumab plus ipilimumab resulted in a higher pCR rate (38% vs. 10%), less viable tumor (median 9% vs. 50%), and greater frequencies of effector, tissue-resident memory and effector memory T cells compared to nivolumab alone [136].

In the CheckMate 9LA trial, nivolumab + ipilimumab + two cycles of chemotherapy exhibited durable survival benefit compared with chemotherapy alone in advanced NSCLC patients with or without brain metastases [137]. The POSEIDON trial reported for the first time that first-line durvalumab + tremelimumab + chemotherapy for metastatic NSCLC patients achieved both the PFS and OS endpoints, with an mPFS of 6.2 months and an mOS of 14 months, compared with 4.8 months and 11.7 months for chemotherapy alone [138].

Combination therapy with PD-1 and CTLA-4 blockers also showed benefit in other cancer types. In the CheckMate 648 trial, first-line use of nivolumab combined with ipilimumab in patients with advanced esophageal squamous cell carcinoma showed an OS benefit over chemotherapy alone. In patients with tumor cell PD-L1 expression of 1% or higher, the OS of nivolumab combined with ipilimumab was significantly longer than that of chemotherapy, with mPFS of 13.7 months and 9.1 months, respectively. Overall survival was also significantly longer with nivolumab plus ipilimumab than with chemotherapy in the overall population [139].The data from CheckMate 204 showed that combination nivolumab plus ipilimumab was efficacious in patients with asymptomatic melanoma brain metastases (MBM). The 36-month intracranial PFS was 54 1%, and OS was 71 9%, supporting first-line use of nivolumab plus ipilimumab. Some patients with symptomatic disease also achieve a long-term response with the combination [140]. Dual PD-1 and CTLA-4 blockade by balstilimab and zalifrelimab combination showed promising and durable clinical activity in patients with recurrent and/or metastatic cervical cancer who relapsed after platinum-based therapy. Compared with the ORR of 4% to 14% for current second-line therapy, the combination therapy achieved an ORR of 25.6%, and the effect was durable, which is very promising. ORR was higher in PD-L1-positive patients and squamous cell carcinoma patients, reaching 32.8% and 32.6%, respectively [141]. Other important clinical trials that assessed the efficacy of dual PD-1 and CTLA-4 blockade include NEOSTAR in NSCLC, CheckMate-214 in renal cell carcinoma, Checkmate-142 in colorectal cancer, CheckMate 067 in Melanoma, CheckMate 040 in hepatocellular carcinoma, CheckMate 743 in malignant pleural mesothelioma and CheckMate 648 in esophageal squamous cell carcinoma.

### Combination therapy with chemotherapy

Chemotherapy was previously thought to directly or indirectly damage CTLs to inhibit the immune system. Recent studies have shown that chemotherapy not only directly kills cancer cells but also positively regulates the immune system to change the local tumor immune microenvironment. For example, chemotherapy induces immunogenic cell death (ICD) [142], promotes the release of tumor antigens and damage-associated molecular patterns (DAMPs) and activates DCs to increase cross-presentation of antigens. In addition, chemotherapy can also induce local production of CXCL10, recruit T cells to the tumor bed [134] and enhance the differentiation of antitumor-specific CTLs [143]. Chemotherapy can also reduce the number of immunosuppressive cells, such as myeloid-derived suppressor cells (MDSCs) [144] and Tregs [145]. In addition to prolonging the efficacy of immunotherapy, tumor shrinkage due to chemotherapy also reduces the risk of drug-resistant clones.

In the past decade, different types of chemotherapeutic drugs have demonstrated the ability to regulate multiple anticancer immune pathways [146]. Given the wide application of chemotherapy in regulating the cancer immune response, combination therapy with ICIs and chemotherapeutic drugs remarkably improves clinical outcomes by enhancing the activity of CTLs. Systemic chemotherapy (SC) has been reported to have a negative immune effect, but local chemotherapy (LC) enhances the immune response. Combination therapy with LC and anti-PD-1 antibodies significantly promoted the immune response and survival rate of glioblastoma. The proliferation of antigen-specific effector T cell clones increases with the upregulated infiltration of cancer-associated DCs in LC-treated mice. In contrast, SC leads to systemic and intratumoral lymphatic exhaustion and reduced immune memory in long-term survivors [147].

Combination therapy with ICB and chemotherapy has been highlighted in the medical field and extensively evaluated in clinical trials, especially for treating advanced NSCLC. To date, combination therapy with ICB and standardized chemotherapy has achieved promising efficacy in NSCLC (KEYNOTE-189, IMpower130), small-cell lung cancer (SCLC, KEYNOTE-407, IMpower133) and triple-negative breast cancer (TNBC, KEYNOTE-355, IMpassion130).

The advantages of combination therapy with ICIs and chemotherapy have been confirmed [130]. In the clinical trial KEYNOTE-021, the efficacy of combination therapy with pembrolizumab and chemotherapy was much higher than that of chemotherapy alone (55% vs. 29%), and it decreased the risk of disease progression by 47% [148].

Breast cancer is the most prevalent malignancy in females, and TNBC is the subtype with the highest recurrence and mortality rates. Hormone therapy and targeted therapy are ineffective for treating TNBC; it is mainly treated with conventional chemotherapy, but the efficacy is not ideal. Despite great advances in immunotherapy, its application to treatment of TNBC is a huge challenge. The combination of nab-paclitaxel and atezolizumab can effectively prolong PFS in patients with metastatic TNBC. However, the combination of paclitaxel and atezolizumab did not meet the primary endpoint of the clinical trial [149, 150]. This shows that the combination of different chemotherapy drugs with ICB may induce different therapeutic effects in the same type of tumor. A systematic analysis of the TME before and after chemotherapy and/or immunotherapy in TNBC patients would help to clarify the therapeutic mechanism and improve its efficacy. Paclitaxel-based chemotherapy may weaken the core anticancer immune cells. However, the activities of those cells can be significantly enhanced by ICIs, suggesting that combination therapy with paclitaxel and atezolizumab influences the efficacy of anti-PD-L1 antibodies in TNBC patients [151]. The dosage form of chemotherapeutic drugs may also influence the efficacy of combination therapy. Paclitaxel nanomicelles can stimulate the antigen presentation of DCs and activate anticancer immunity. Combination therapy with low-dose paclitaxel nanomicelles and anti-PD-1 antibodies enhanced therapeutic efficacy by inducing CD8^+^ T cell-dependent anticancer immunity [152].

The combination of immunotherapy with chemotherapy also showed potential advantages in clinical trials compared to immunotherapy alone.

The CheckMate-816 phase III clinical trial showed that nivolumab plus adjuvant chemotherapy can markedly improve event-free survival in patients with resectable NSCLC, meeting its primary endpoint. Nivolumab plus chemotherapy exhibited a statistically significant clinical improvement in event-free survival compared to that of neoadjuvant chemotherapy alone.

In the KEYNOTE-407 (NCT02775435) randomized trial, pembrolizumab plus chemotherapy substantially improved OS and PFS in NSCLC patients compared to that of placebo plus chemotherapy [153].

Among the clinical trials on perioperative immunotherapies for NSCLC patients, IMpower010 is the first phase 3 clinical trial demonstrating that immunotherapy can considerably improve DFS in patients with early stage resectable NSCLC compared with best supportive care after adjuvant platinum-based chemotherapy. Based on this clinical trial, atezolizumab has been approved by the FDA as an adjuvant therapy for stage II-IIIA NSCLC patients whose tumors expressed PD-L1 ≥ 1% after surgery and platinum-based chemotherapy [154].

The NADIM trial showed that combination of neoadjuvant nivolumab with platinum-based chemotherapy is feasible in patients with resectable stage IIIA NSCLC. Neoadjuvant immunotherapy combined with chemotherapy has the potential to change some stage III NSCLC to curable disease [155].

Immunotherapy plus chemotherapy has demonstrated good therapeutic efficacy in unresectable locally advanced NSCLC. Five-year follow-up data from the PACIFIC study showed that the combination of durvalumab after chemoradiotherapy significantly improved OS and PFS in patients with unresectable stage III NSCLC. The KEYNOTE-799 trial showed that the combination of pembrolizumab plus chemoradiotherapy has promising anti-tumor activity in patients with unresectable, locally advanced, stage III NSCLC [156]. The results of the GEMSTONE-301 trial showed that consolidation therapy with sugemalimab has superior efficacy and acceptable side effects in patients with stage III NSCLC after concurrent or sequential chemoradiotherapy.

The results of the KEYNOTE-811 trial showed that the addition of anti-PD-1 antibodies to the conventional treatment regimen (trastuzumab plus chemotherapy) for HER2-positive gastrointestinal malignancies improved therapeutic efficacy with a longer duration of survival [157]. In addition, the adverse effects of this new treatment regimen (anti-PD-1 antibody + trastuzumab + chemotherapy) are also completely manageable. Based on these data, this new treatment regimen has been approved by the FDA as a first-line intervention for the treatment of HER2-positive gastric and gastroesophageal adenocarcinomas (GEACs).

However, due to the high heterogeneity of the TME, the overall efficacy of immunotherapy on tumors remains low, and the combination of immunotherapy and other therapies does not produce synergistic effects in all patients. A retrospective analysis of three cohorts of patients with advanced GEAC found that in patients with low PD-L1 expression in the tumor, compared with chemotherapy alone, the combination of chemotherapy and PD-L1 inhibitor had no significant effect on OS and PFS [158].The results of the GEMSTONE-302 trial on sugemalimab in the treatment of stage IV NSCLC showed that sugemalimab plus chemotherapy provided significant and clinically meaningful PFS improvement in different subtypes of metastatic NSCLC regardless of PD-L1 expression, with a 52% reduction in the risk of disease progression and death [159]. Additionally, the combination therapy showed some benefit to OS, with a 2-year survival rate of 47.1%.

The CHOICE-01 trial showed that the combination of toripalimab with first-line standard chemotherapy resulted in longer PFS, higher objective response rate (ORR) and longer duration of relief in patients with advanced NSCLC with manageable side effects [160]. In the IMpower132 trial, the addition of atezolizumab to the combination of pemetrexed + platinum complexes showed improved PFS in patients with stage IV nonsquamous NSCLC [161].

### Combination therapy with radiotherapy

Radiotherapy is a local treatment that directly kills cancer cells with radiation. In addition, it triggers an antitumor immune response by releasing tumor-associated antigens, inducing type I interferons (IFNs) and changing the immunosuppressive TME. Immunotherapy aims to kill cancer cells and lesions by utilizing the immune system. Notably, the immunogenicity induced by radiotherapy in the body can stimulate the release of cellular contents, thus producing in situ vaccines, which is conducive to the anticancer effect. As a novel therapeutic strategy, combination treatment with radiotherapy and immunotherapy has achieved good clinical outcomes. To date, more than 100 clinical trials have been conducted to analyze the efficacy of combination treatment with radiotherapy (radioconjugates) and immunotherapy.

It has been reported that radiotherapy markedly upregulates the cell adhesion factors ICAM-1 and VCAM-1 on the surface of cancer cells. Combination treatment with radiotherapy and anti-PD-1 antibodies activates tumor-specific T cells in the TME [162]. In addition, combination treatment with radiotherapy and anti-PD-L1 antibodies increases the infiltration of CD8^+^ T cells and reduces the accumulation of MDSCs and regulatory T cells, thereby improving anti-tumor immunity [163]. Preclinical studies have shown that the addition of immunotherapy can achieve higher local control rates at the same radiation dose [164]. A recent preclinical study demonstrated that radiotherapy facilitates immunotherapy in NSCLC by activating certain types of club cells. These cells subsequently release proteins that alleviate the inflammatory response and enhance the antitumor immune response by effectively inhibiting MDSCs, thereby significantly enhances the therapeutic efficacy of PD-1 inhibitors [165]. The authors compared the efficacy of hypofractionated radiotherapy (different hypofractionated doses of radiotherapy) + PD-1 inhibitor and PD-1 inhibitor monotherapy in mouse NSCLC models and found that the tumor-free survival rate of mice in the combination group was 4 times that of the monotherapy group (40% vs. 10%). In addition, the secreted protein CC10 is thought to be a biomarker for the efficacy of combination treatment with radiotherapy and immunotherapy. Interestingly, the gene *Scgb1a1*, which encodes CC10, is a biomarker for radiotherapy-activated club cells.

Local radiotherapy (RT) induces an antitumor immune response partially by activation of immune evasion and tissue remodeling processes, e.g., via upregulation of PD-L1 and TGF-β expression. A combination treatment with Bintrafusp alfa (BA) and RT (BART) enhanced tumor infiltrating white blood cells, reprogramed the TME and reduced radiotherapy-induced fibrosis, leading to reconstitution of immune TME and spontaneous lung metastasis regression. Combination treatment with BART may further support clinical transformation by eradicating cancer lesions while preserving normal tissues [166].

Clear clinical evidence indicated that colon cancer patients with mismatch repair defects (MMRd) or those with microsatellite instability-high (MSI-H) respond well to immunotherapy, but the vast majority of microsatellite-stable (MSS) patients do not benefit from immunotherapy [167]. PDAC is one of the cancers most resistant to immunotherapy. So far, immunotherapy alone has been proved to be ineffective for the treatment of PDAC patients, so it is necessary to carry out combination therapy based on the mechanism of intrinsic resistance to immunotherapy [168]. A phase II trial study found that radiation therapy enhances the response to immunotherapy with ipilimumab and nivolumab in patients with MSS CRC and PDAC [169].

Recent studies have suggested that combination treatment with radiotherapy and anti-PD-1/anti-PD-L1 antibodies has better therapeutic efficacy compared with that of anti-PD-1/anti-PD-L1 antibodies monotherapy. Conventional fractionated radiotherapy, hypofractionated radiotherapy and stereotactic body radiation therapy (SBRT) have been applied in combination with radiotherapy and immunotherapy in advanced NSCLC patients, with radiotherapy administered prior to immunotherapy. The PEMBRO-RT clinical trial was the first to explore the efficacy of pembrolizumab as a maintenance therapy following SBRT in advanced lung cancer patients, which double that of the placebo group (41% vs. 19%) with an acceptable tolerance [170]. A phase I clinical trial recruited patients with advanced solid tumors that had progressed after standard therapy and were given nivolumab + urelumab or nivolumab + cabiralizumab concurrently with and after SBRT. The results showed that 2 patients achieved complete response (5%), 7 patients exhibited partial response (17%), 12 patients showed stable disease (29%), 20 patients had disease progression (49%), mPFS and mOS were 3.0 months and 17.0 months, respectively. Patients with elevated serum IL8 prior to SBRT did not respond to treatment. These data demonstrate that SBRT in combination with nivolumab + urelumab or nivolumab + cabiralizumab is feasible in advanced solid tumors with modest antitumor activity and acceptable toxicity [171].

It remains unclear what is the optimal time for combination treatment with ICIs and radiotherapy to maximize the efficacy and minimizing the AEs in different types of cancers, which merits further investigation.

### Combination therapy with surgery

Under normal circumstances, tissue damage caused by surgery can trigger an inflammatory response and the transformation to a Th2 immune response, involving enhanced activity of Tregs and expansion of MDSCs. Surgical stress results in dysfunction of NK and T cells. Therefore, the perioperative period is critical to enhance immunity and reduce cancer recurrence. Neoadjuvant therapy can preoperatively shrink cancer lesions, reduce surgical difficulty and resect micrometastases to decrease the risk of recurrence. This therapy may even be beneficial for patients who are unable to be surgically treated. A phase II clinical trial, CheckMate-159 (NCT02259621), showed the safety of preoperative neoadjuvant immunotherapy with nivolumab in NSCLC patients; this treatment was associated with fewer AEs, did not delay surgical treatment and caused a major pathological response in 45% of excised tumors. Intraoperative pathology also confirmed the massive infiltration of T cells and macrophages in cancer sections, suggesting that preoperative ICIs can enhance the antitumor immune response [172]. The clinical trial NADIM (NCT03081689) on stage IIIa lung cancer patients showed that after preoperative neoadjuvant treatment with nivolumab + carboplatin + paclitaxel, the main pathological response (MPR), pathological complete remission (pCR) and partial remission (PR) in imaging examinations achieved 85.36%, 71.4% and 72%, respectively [173].

Taken together, these results indicate that neoadjuvant therapy with ICIs achieves extraordinary outcomes, although its efficacy needs to be validated in multicenter large-scale clinical trials. In addition, imaging-based response was significantly later than pathological response, which is a huge obstacle for the conventional preoperative imaging. At present, multiple clinical trials on neoadjuvant immunotherapies for NSCLC are ongoing, including NCT02938624, NCT03217071, NCT02818920 and NCT02259621, which are expected to provide more data.

### Combination therapy with targeted drugs

#### Combination therapy with EGFR-TKI

EGFR mutations are the most common malignant drivers of lung cancer. NSCLC patients with EGFR mutations express PD-L1 at varying levels, and as a result, the therapeutic efficacy of combination treatment with EGFR inhibitors and ICIs remains controversial [174, 175]. EGFR mutations activate transcription factors such as STAT3, STAT1 and NF-κB, which further translocate into cell nuclei to induce PD-L1 expression. In addition to EGFR, PD-L1 can also be influenced by *TP53, KRAS, STK11* and other genes. Tyrosine kinase inhibitors (TKIs) enhance immune presentation by upregulating MHC class I and II molecules [176]. TKIs can enhance CTL-mediated anticancer activity, inhibit apoptosis of T cells and stimulate the production of IFN-γ [177]. Moreover, TKIs reduce the infiltration of Tregs in the TME by accelerating Foxp3 degradation [178]. Due to the immunomodulatory effects of TKIs, combination treatment with TKIs and immunotherapy is considered a promising strategy, although current findings are ambiguous.

However, several clinical trials have suggested that neither ICI monotherapy nor combination treatment with TKIs and ICIs is recommended for lung cancer driven by gene mutations due to the low efficacy, high incidence of AEs and rapid progression of diseases. The efficacy of PD-1 inhibitors in advanced NSCLC patients with EGFR/ALK mutations is generally lower than 5%, while that of targeted therapy is up to 70% [174]. A retrospective study in Japan involving more than 20,000 advanced lung cancer patients with EGFR mutations found that the overall incidence of interstitial pneumonia or immune pneumonia was 4.8%: 4.6% in targeted monotherapy, 6.4% in ICI monotherapy and 25.7% in combination treatment with ICIs and TKIs [179]. A number of clinical trials on TKIs were discontinued due to poor efficacy and severe AEs. A recent clinical trial analyzed immunotherapy efficacy and genetic data in 155 cancer patients. Of these patients, 2 lung adenocarcinoma patients with EGFR mutations developed drug resistance after chemotherapy and EGFR-TKI medication, and they further suffered rapid progression after switching to the PD-1 inhibitor nivolumab, with significant enlargement of cancer lesions by 53.6% and 125%, respectively [85]. Representative clinical trials using PD-(L)1 inhibitor in EGFR-mutant NSCLC include KEYNOTE-010 and CheckMate 012 for PD-1 inhibitor monotherapy, and NCT02088112, TATTON, NCT01998126 for combination treatment with PD-(L)1 inhibitor and TKIs.

EGFR T790M mutation-negative and KRAS/TP53 comutation NSCLC patients were responsive to combination treatment with targeted therapy and ICIs, which may be attributed to the higher incidence of coexisting high expression levels of PD-L1 (≥ 10%), high percentage of CD8^+^ tumor infiltrating lymphocytes (TILs) (20% vs. 4%) and lower frequency of FOXP3^+^ TILs in EGFR T790M mutation-negative NSCLC patients compared to those in EGFR T790M mutation-positive patients [180]. The relatively high TMB in NSCLC patients with KRAS mutations may be a potential explanation of their good response to ICIs, while that in patients carrying other key driver gene mutations (e.g., EGFR, ALK, ROS1) was relatively low [181].

Thus, targeted therapy is still preferred for EGFR-mutant lung cancer patients. The efficacy and safety of combination treatment with ICIs and TKIs in lung cancer patients carrying driver gene mutations remain uncertain and should be further analyzed to identify specific populations that may benefit from it.

#### Combination therapy with agonists of the STING pathway

MYC binds to the DNMT1 promoter and activates its transcription, thereby inhibiting the cGAS-STING pathway through epigenetic regulation [182]. The cGAS-STING pathway is vital in linking innate immunity and adaptive immunity against cancers [183]. Cancer cells can escape immune surveillance by inhibiting the cGAS-STING pathway [182]. The cytosolic DNA-sensing cGAS-STING pathway has therefore been widely analyzed in immune activation [184, 185].

The surface expression of PD-L1 can be upregulated by targeting the DNA damage response (DDR) protein poly ADP-ribose polymerase (PARP) and checkpoint kinase 1 (CHK1) [186]. PARP is a DNA repair enzyme, and its inhibitors (PARPis) significantly upregulate PD-L1 [187], which activates the STING/TBK1/IRF3 pathway, upregulates chemokines such as CXCL10 and CCL5 [94] and induces the activation of CTLs [188]. DDR protein inhibitors also upregulate chemokines such as CXCL10 and CCL5 by activating the STING/TBK1/IRF3 pathway, thereby inducing the activation of CTLs [188].

By promoting the accumulation of cytosolic DNA fragments, PARPis induce antitumor immunity independent of BRCAness by activating the DNA-sensing cGAS-STING pathway and stimulating the production of type I interferons. ICB further enhances the regulatory effects of PARPis [189]. Therefore, PARPis are promising immunomodulators for ICB in cancer treatment.

Remarkable results have been achieved in the maintenance treatment of recurrent ovarian cancer and breast cancer with PARPis combined with ICIs [190–192]. At present, four PARPis have been approved by the FDA, including olaparib, rucaparib, talazoparib and niraparib. SCLC, which is highly sensitive to platinum-based chemotherapy, usually express high levels of PARP1, suggesting the important role of DNA damage repair [193]. A phase II randomized clinical trial showed that combination treatment with the PARPi veliparib and standard chemotherapy achieved an ORR of 39% in SCLC patients [194]. Other representative clinical trials combining PARPi and PD-(L)1 inhibitors include TOPACIO/Keynote-162, NCT04681469, NCT04837209, NCT03824704, NCT02873962, NCT03694262, NCT03737643, NCT03642132 and NCT03598270. Currently, the application of PARPis combined with ICIs in the treatment of SCLC is in its infancy, and the specific mechanisms need further investigation. In addition, combination treatment with ICB and inhibitors of DDR, ATR, ATM, CHK1 and MK2 requires in-depth examination.

#### Combination with other targeted therapy

ICB with anti-G-CSF antibodies and Src inhibitors is capable of blocking neutrophil infiltration, thereby preventing pY696-EZH2-driven brain metastases. EZH2 is upregulated in brain metastases and phosphorylated at tyrosine 696 by Src tyrosine kinase, which changes its binding preference from histone H3 to RNA polymerase II and switches EZH2’s function from a methyltransferase to a transcription factor responsible for upregulating c-JUN. Upregulation of c-JUN further triggers the activation of carcinogenic inflammatory cytokines such as granulocyte-colony stimulating factor (G-CSF), which accelerates brain metastases by recruiting Arg1-positive and PD-L1-positive immunosuppressive neutrophils into the brain [195]. The therapeutic efficacy of combination treatment with anti-G-CSF antibodies or ICB for treating brain metastases has been verified in multiple mouse models.

PGE2 driven by cyclooxygenases is produced by various types of cancers and consequently induces malignant growth by escaping type I interferon and/or T cell-induced eradication of cancer cells. The synergistic effect of cyclooxygenase inhibitors combined with ICB has been proven to significantly induce cancer cell eradication [196].

Combination treatment with ICB and MDSC-targeted therapy in primary and metastatic castration-resistant prostate cancer (CRPC) presents a strong synergistic response by upregulating interleukin-1 (IL-1) receptor antagonists and inhibiting proinflammatory cytokines released by prostate cancer cells [197].

Reasonable sequencing assists in overcoming innate and acquired drug resistance following combination treatment with PD-1/PD-L1 inhibitors and MAPK-targeted therapy. Clinical benefits obtained from MAPK inhibitors (MAPKis) are linked with prior ICI treatment. Anti-PD-1/PD-L1 antibody lead-in before MAPKi treatment not only inhibits melanoma brain metastasis (MBM) but also enhances the survival rate of mice through the potent clonal expansion of T cells in intracranial and extracranial metastasis sites [198].

The KEYNOTE-775 phase III clinical trial showed that patients with advanced endometrial cancer treated with the anti-PD-1 monoclonal antibody Pembrolizumab plus the oral multi-receptor TKI Lenvatinib exhibited significant improvements in OS and PFS compared to that seen with chemotherapy alone. The median PFS (7.2 months vs. 3.8 months) and median OS (18.3 months vs. 11.4 months) of the Pembrolizumab/Lenvatinib group were significantly higher than that for the chemotherapy group.

Studies have shown that intermittent PI3K inhibition can attenuate the inherent immunosuppressive activity of Pten-null cancer cells and transform cold tumors into a state of high T cell infiltration, paving the way for successful immune checkpoint therapy [199].

Pattern recognition receptors (PRRs) are molecules central to initiating and maintaining innate immunity and which include TLRs, the RGR family and cGAS-STING; they monitor local infection and/or tissue damage, thereby preventing systemic infection the production of malignant cells. TLRs are the best-studied PRRs and central to the activation of the innate immune response. TLRs agonists are a major direction for anti-tumor immunotherapy. In addition, as TLRs agonists activate innate immunity and are the cornerstone of activation of the adaptive immune response, they have an inherent advantage when combined with anti-PD-(L)1 therapy.

Intratumoral immunotherapy using TLR agonists aims to induce or enhance local tumor inflammation and immunity by mimicking intracellular microorganisms (viruses or bacteria), thereby evoking cytotoxic CD8 + T cell responses, promoting the infiltration of TILs, and stimulating CD4 + T cells to produce effector molecules such as IFN-γ, which in turn enhances the anticancer effects of anti-PD-1 antibodies. In addition, the use of TLR agonists as vaccine adjuvants is also a direction of future development. However, as the systemic administration of TLR agonists may lead to systemic inflammation and treatment-related side effects, current clinical development has focused on local intratumoral injection to localize inflammation to the tumor [200, 201].

### Combination therapy with anti-angiogenic drugs

Local hypoxia and low pH levels caused by the abnormal structure and function of tumor blood vessels result in an inhibitory tumor immune microenvironment. Hypoxia triggers the accumulation of MDSCs and accelerates the differentiation of tumor-associated macrophages (TAMs) into immunosuppressive M2 macrophages [202]. In addition, hypoxia indirectly stimulates the aggravation of Tregs by upregulating CC chemokine ligands. It also upregulates PD-L1 expression in cancer cells and TIM-3 and CTLA4 expression in TAMs, MDSCs and Tregs and indirectly upregulates PD-1 expression in CD8^+^ T cells, thus inhibiting the activation of immune cells. The increased tumor vascular permeability and decreased lymphatic vessels contribute to the high tumor interstitial fluid pressure (TIFP), which hinders immune effector cells from entering the cancer lesion [203].

Anti-angiogenic drugs reprogram the TME by normalizing immature blood vessels and reducing the activities of immunosuppressive cells such as MDSCs and Tregs [204]. T cells that bind to tumor antigens are more effectively activated through blocking VEGF-induced inhibition of DC maturation. The normalized tumor vascular structure is favorable to the infiltration of CTLs into cancer lesions. However, high-dose antiangiogenic drugs result in excessive vascular pruning, which further exacerbates the hypoxia and acidosis of the TME. In addition, high-dose anti-VGEF drugs can also accelerate the deposition of ECM, local hypoxia and immunosuppression [204]. However, low-dose vascular endothelial growth factor inhibitors can reduce the sprouting of immature blood vessels and make them structurally and functionally normal, facilitating the delivery of chemotherapy drugs and promoting the infiltration of killer T cells into tumors [205].

Anti-angiogenesis therapy promotes the intratumoral infiltration of PD-1^+^ Tregs. There are two types of TAMs. They are derived from monocytes or alveolar cells. The former type relies on CSF-1R, and the latter is sensitive to cisplatin and contributes to the establishment of a TGF-β-rich TME. Dual inhibition of TAMs with CSF1R inhibitors and cisplatin suppresses Tregs, which redirect anti-PD-1 antibodies to CD8^+^ T cells. As a result, immunotherapy with antiangiogenic drugs exerts an excellent efficacy to eradicate cancer lesions in most cases [206].

Anti-angiogenic therapy can enhance the efficacy of immunotherapy by downregulating immunosuppressive factors during tumor angiogenesis and reversing the de-energized state of endothelial cells [207]. As a malignant ecosystem, the TME is composed of “normal cells” that behave extremely abnormally in addition to cancer cells. Endothelial cells in tumor vasculature are a good example. Despite its abundant vasculature, the tumor is still highly hypoxic due to the abnormal function and structure of these blood vessels. Some multi-target TKIs can simultaneously inhibit fibroblast growth factor receptors (FGFR) and platelet-derived growth factor receptors (PDGFR), thereby reducing the activity of cancer-associated fibroblasts [208]. How to combine targeted therapy that brings “normalized microenvironment” with immunotherapy to exert excellent efficacies in cancer patients still needs to be explored.

The clinical trial IMpower150 assessed the efficacy and safety of combination treatment with atezolizumab and bevacizumab/chemotherapy on newly treated stage IV nonsquamous NSCLC. Compared with bevacizumab + carboplatin + paclitaxel, the addition of atezolizumab to the above regimen presents controllable side effects and satisfactory anticancer activity, which provides a novel option for treating nonsquamous NSCLC patients [209]. The phase 1a/b JVDF clinical trial explored the efficacy of ramucirumab combined with pembrolizumab on advanced NSCLC and found it achieved an ORR of 30% with controllable side effects [175]. Taken together, these studies suggest that combination treatment with ICIs and antiangiogenic drugs is a promising strategy, and its efficacy, safety and mechanisms should be further analyzed.

### The influence of dietary composition on immunotherapy

The effect of diet composition on immunotherapy has shown broad importance in cancer treatment.

Vitamin C is an electron donor involved in the biochemical reactions of cancer stem cells and the synthesis of collagens and hypoxia-inducible factors, which are important for metastasis as they regulate ECM reprogramming [210]. Specific doses of vitamin C are able to prevent glycolysis in cancer cells as well as the synthesis of nitroso groups, indicating the importance of this vitamin for cancer treatment [210]. Recent studies have shown that vitamin C indirectly enhances the anticancer immune response of anti-PD-L1 antibodies [211]. High-dose vitamin C regulates the infiltration of immune cells in the TME and delays malignant growth in a T cell-dependent manner. Vitamin C not only enhances the cytotoxic activity of adoptively transferred CD8^+^ T cells but also has promoted the therapeutic efficacy of immune checkpoint therapy (ICT) [212]. The synergistic effect of vitamin C and anti-PD-1 antibodies has been validated in mouse models of lymphoma [211, 213]; it enhances the intratumoral infiltration of CD8^+^ T lymphocytes, macrophages, DCs and NK cells and upregulates the expression of granzyme B and IL-12 [211].

Stimulator of interferon genes (STING) agonists derived from the microbiota regulate macrophage polarization and NK cell-DC crosstalk by inducing the production of type I interferon (IFN-I) in intratumoral monocytes. Microbiota modulation with a high-fiber diet enhances anticancer the efficacy of ICB by triggering the IFN-I/NK cell/DC cell axis [214].

Ketogenic diet is becoming popular. A recent study reported that energy change induced by a ketogenic diet enhanced the efficacy of anti-CTLA-4 immunotherapy by downregulating PD-L1 expression and upregulating expression of IFN-I and antigen presentation genes. The activated AMPK pathway is responsible for phosphorylating PD-L1 at Ser283, which in turn disrupt its interaction with CMTM4 and degrades PD-L1. Moreover, activated AMPK also represses PRC2 by phosphorylating EZH2 and eventually upregulates the expression of IFN-I and antigen presentation genes [202].

The recently proposed fasting/fasting-mimicking diet reduces the survival of cancer stem cells and delays the progression of TNBC by inhibiting the activity of glucose-dependent protein kinase A. In differentiated tumor cells, the activation of starvation escape signaling pathways can be blocked using certain inhibitors to inhibit tumor progression and improve patient outcomes [215].

Alcohol consumption induces ALDH2 and subsequently upregulates PD-L1 expression in CRC, thereby protecting it from immune surveillance. Therefore, the combination of ALDH2 inhibition and anti-PD-1 therapy enhances the anti-tumor immunity and can be used as a novel strategy to enhance the efficacy of ICB in CRC patients, especially those who consume alcohol [216].

### Combination therapy with TIL adoptive cell therapy

Although great progress have been made in utilizing ICB for treating NSCLC, a considerable number of NSCLC patients do not benefit from the treatment. Additionally, its efficacy in combination treatment is far from satisfactory. A relevant study reported that most NSCLC cases relapsed within 12 months of combination treatment with ICB and platinum-based chemotherapy [217]. Notably, some NSCLCs are cold tumors that lack activated tumor-specific T cells, which is a vital reason of primary resistance to ICB. More effective combination treatment regimens are needed to turn the cold advanced NSCLC into hot tumors. Some studies proposed that adoptive cell therapy (ACT) using the patient's own T cells may be ideal for regulating the TME.

A previous study demonstrated that some melanoma patients benefitted from ACT using TILs extracted from tumor tissue from patients [218]; this therapeutic strategy has also been reported to be effective in treating cholangiocarcinoma [219], cervical cancer [220], colorectal cancer [221] and breast cancer [222]. A recent phase 1 clinical trial (NCT03215810) was the first study to analyze the efficacy of TILs combined with nivolumab in advanced NSCLC patients and found that 2 patients achieved sustained complete remission 1.5 years later [223].

### Combination with cell therapy

The combination of PD-1 blockade and third-generation anti-GD2-CAR-T cell therapy produced robust responses in melanoma patients [224]. A preclinical study showed that CAR-T cell therapy targeting PD-1-blocking scFv improves the viabilities of tumor-specific T cells. The scFv secreted by CAR-T cells are localized in the tumor, which may prevent the cytotoxicity associated with systemic checkpoint inhibition [225].

### Combination with oncolytic virus therapy

Oncolytic virus therapy can increase the activities tumor-specific effector and memory T cells that attack tumor cells [226, 227]. Therefore, oncolytic virus therapy is also considered a type of immunotherapy. Engineered oncolytic viruses recombinantly expressing monoclonal antibodies against the immunosuppressive molecule TIGIT have been constructed in a previous study. These recombinant oncolytic viruses could turn the “cold” TME to “hot” and induce an effective anti-tumor immune response [228]. In addition, combination of these viruses with PD-1 inhibitors or LAG-3 inhibitors resulted in better efficacy and caused tumor regression.

A novel combination of the colony-stimulating factor 1 receptor (CSF-1R) inhibitor PLX3397, oncolytic viruses and anti-PD-1 antibodies has been analyzed and significantly controls malignant growth and prolongs the survival of colorectal cancer (CRC) mouse models. Approximately 43% and 82% of CRC mice implanted with CT26 and MC38 cells survived long-term after the triple combination treatment, respectively, which can be attributed to reprogrammed antitumor immunity by enhancing T cell infiltration and CD8^+^ T cell function [229].

### Combination with mechanical immune checkpoint blockade

In addition to traditional immune checkpoints, one study have proposed the concept of mechanical immune checkpoints, which can be used for developing a new generation of targeted therapies, thereby improving the efficacy of cancer immunotherapy [230]. The study found that cancer-cell stiffening could serve as a mechanical immune checkpoint. By depleting the cholesterol level in the plasma membrane of tumor cells to increase the stiffness of cancer cells, the cytotoxicity against stiffened cancer cells can be augmented, and the effect of adoptive T cell therapy can be improved.

### Combination with immunomodulatory vaccines

A phase I/II clinical study showed that the combination of nivolumab and IO102/IO103, an investigational vaccine targeting indoleamine 2, 3-dioxygenase (IDO) and PD-L1, showed an ORR of up to 80% in metastatic melanoma patients [231]. This combination of immunomodulatory vaccine with PD-1 inhibitor significantly reduced tumor burden and increased the PFS to 26 months.

### The effects of circadian rhythm on the efficacy of immunotherapy

A recent study found for the first time an evident correlation between the body's biological clock and circadian rhythm and the efficacy of immune checkpoint inhibitors. If at least 20% of the dose was infused after 16:30 pm during treatment, the patient's risk of death was doubled, and the 5-year survival rate was also reduced by 20% [232]. Some small-scale clinical studies showed that the immune response activated by the injection of a vaccine between 09:00 and 11:00 a.m. was significantly better than that injected between 15:00 and 17:00 p.m. [233]. Cytokine immunotherapy with recombinant human IL-2 injections also seems to exhibit differences in efficacy at different times of day [234].

### Combination with DNA damage response (DDR)-targeted therapy

Immunotherapy has revolutionized cancer treatment and dramatically improved the outcomes in patients with multiple tumor types. However, most patients still do not benefit from these treatments, especially those lacking pre-existing T cell infiltration. Loss of DDR is a major determinant of tumor immunogenicity. Growing evidence supports the following roles of DDR-targeted therapy in tumor immunity [235]: (1) promoting antigenicity by increasing mutability and genomic instability, (2) enhancing adjuvanticity by activating cytosolic immunity and immunogenic cell death and (3) favoring reactogenicity by modulating of factors that control the tumor-immune cell synapse.

### Combination with inhibition of M2 macrophages

Histamine from allergic reactions can activate macrophages and inhibit the anti-tumor immune response of T cells, thereby causing resistant to PD-1 inhibitors. HRH1-activated macrophages polarize to an M2-like immunosuppressive phenotype and increased expression of the immune checkpoint VISTA, leading to T cell dysfunction [236]. H1-antihistamines can effectively reverse the immunosuppressive effects of M2 macrophages, thereby restoring T cell activity and the therapeutic efficacy of anti-PD-1/CTLA-4 treatment. Targeting HRH1 and VISTA may identify powerful combination therapies to overcome ICB resistance.

## Improvement of ICI efficacy by regulating the expression of PD-L1

In addition to combination treatment with ICIs, other interventions that influence the expression of PD-L1 also affected the efficacies of PD-(L)1 blockade. PD-L1 is upregulated on the surface of many types of cancer cells by IFN-γ and TNF-α, and the regulation involves some endogenous carcinogenic pathways (e.g., the PI3K-AKT and AMPK pathways). Upregulated PD-L1 assists cancer cells in immune escape by negatively regulating antitumor immunity after binding to PD-1 [174]. Altered PD-L1 expression (either through upregulation or downregulation) yields better efficacy in combination with immunotherapy. After downregulation of PD-L1 expression, the inhibited PD-L1/PD-1 axis releases the brake on the immune system. In contrast, upregulated PD-L1 turns cold tumors into hot tumors; therefore, the PD-L1/PD-1 axis might have more power to inhibit the anti-tumor immune system. Targeting this pathway can also produce good therapeutic effects.

Targeting PD-L1 regulation can also produce good therapeutic effects. A multistage sensitive nanocomplex (MUSE) loaded with PD-L1/CD47 multiple targeting CRISPR/Cas9 system was developed for coactivation of both T cells and macrophages-mediated antitumor immune response [237]. The prepared MUSE has some beneficial characteristics, including prolonged blood circulation, rapid response to the MMP-9-rich TME, enhanced lysosomal escape, rapid nuclear localization and high transfection efficiency. With these advantages, MUSE loaded with MT-CRISPR/Cas9 demonstrated effective elimination of PD-L1 and CD47 in tumor cells and activated both innate and adaptive antitumor immunity, thereby significantly improving overall survival in mouse model of melanoma with no detectable off-target effects. This study provides new avenues for the development of anticancer treatment regimens and paves the way for CRISPR-based anticancer therapies in the future.

### Signaling pathways for regulating the expression level of PD-L1

Several factors have been found to abnormally enhance PD-L1 expression, including genomic alterations, constitutive activation of oncogenic pathways (e.g., activation of EGFR, mTOR, PI3K, AKT and AMPK pathways and deficiency of PTEN) [175–179] and exogenous factors (e.g., IFN-γ, TGFβ1, TNF-α and IL-17) [85, 179–181].

### Factors that regulate the expression level of PD-L1

Many factors affect the expression levels of PD-L1 in the TME and circulation and thus can affect the efficacy of ICB. Here, we categorize these factors as endogenous factors, signaling pathway changes and external factors. Table 3 summarizes representative preclinical studies of influencing factors. From these studies, we can see that the expression of PD-L1 is complexly regulated. In addition, most studies provide strategies to exploit the expression changes of PD-L1 to enhance the therapeutic effect of ICB.

**Table 3:** Factors that affect PD-L1 expression

Modulator	Effect of the modulator on PD-L1 expression	Tumor model	Combined immunotherapy	Mechanism and results	References
Ketogenic diet	Downregulation	TNBC and CRC	anti-CTLA-4 therapy	1. Energy stress or ketogenic diet treatment decreases PD-L1 protein abundance 2. AMPK phosphorylates PD-L1 at Ser283 to disrupt its interaction with CMTM4 3. AMPK enhances IFNs and antigen-presentation gene expression via repressing PRC2 4. AMPK agonists or ketogenic diets enhance the efficacy of anti-CTLA-4 immunotherapy	[202]
Metformin	Downregulation	TNBC	anti-CTLA-4 therapy	1. Metformin enhances antitumor CTL immunity by blocking PD-L1/PD-1 axis 2. Metformin-activated AMPK directly binds to and phosphorylates PD-L1 at S195 3. Abnormal PD-L1 glycosylation induced by pS195 leads to PD-L1 degradation by ERAD 4. Combination therapy with metformin and anti-CTLA4 has a synergistic antitumor effect	[203]
Ruxolitinib	Downregulation	GBM	MV-s-NAP-uPA + anti-PD-1 therapy	1. Infection with MV-s-NAP led to PD-L1 upregulation and an increase in the levels of MHC class I molecules in glioma cells 2. Intratumoral MV-s-NAP overcomes resistance and synergizes with anti-PD1 blockade 3. Localized MV-s-NAP-uPA infection with systemic anti-PD1 blockade leverages abscopal therapeutic effect 4. Pharmacological inhibition of the JAK1/JAK2 signaling pathway with ruxolitinib improves GBM cure rates by abrogating PD-L1 expression on MDSCs	[238]
mTOR inhibitor (rapamycin)	Downregulation	NSCLC	anti-PD-1 therapy	1. The activation of AKT and mTOR is associated with PD-L1 expression in NSCLC cell lines that harbor a wide spectrum of driver mutations 2. Inhibition of PI3K, AKT or mTOR decreases PD-L1 expression in NSCLC cell lines. Rapamycin decreases PD-L1 expression in murine lung tumors 3. EGF and IFN-γ increase PD-L1 expression through activation of mTOR 4. The combination of rapamycin and a PD-1 blocking antibody decreases lung tumor growth	[175]
CSN5 inhibitor	Downregulation	TNBC	Anti-CTLA-4 therapy	1. TNF-α stabilizes cancer cell PD-L1 in response to chronic inflammation 2. Activation of NF-kB by TNF-α induces CSN5 expression that lead to PD-L1 stabilization 3. CSN5 suppresses the activities of T cell via PD-L1 deubiquitination 4. Destabilization of PD-L1 by CSN5 inhibitor curcumin benefits anti-CTLA4 therapy	[239]
Copper-chelating drugs (Dextran–Catechin and TEPA)	Downregulation	Neuroblastoma	Not applicable	1. Copper transporter 1(CTR-1) and PD-L1 expression in cancer is positively correlated 2. Intracellular Cu levels influence PD-L1 expression in cancer cells 3. Dextran–Catechin and TEPA downregulate PD-L1 expression by inhibiting EGFR and STAT phosphorylation 4. Cu-chelation enhances infiltration of anticancer immune cells and improves the survival of mice with neuroblastoma by downregulating PD-L1	[179]
Epigallocatechin gallate (EGCG)	Downregulation	NSCLC	Not applicable	1. EGCG reduced PD-L1 expression via inhibition of the JAK2/STAT1 pathway 2. EGCG partially restored T cell activity by suppressing PD-L1/PD-1 signaling 3. Oral administration of green tea extract reduced PD-L1-positive cells and inhibited tumor growth in the lungs of NNK treated A/J mice 4. EGCG downregulation EGF-induced PD-L1 through inhibition of Akt phosphorylation in Lu99 cells	[240]
Pin1 inhibitor (ATO, ATRA, Sulfopin)	Upregulation	Pancreatic cancer	Pin1 inhibitor + anti-PD-1 antibody + gemcitabine	1. Pin1 is overexpressed in both PDAC cells and CAFs and correlates with poor prognosis of patients 2. Pin1 inhibition disrupts the desmoplastic and immunosuppressive TME by affecting CAFs 3. Pin1 inhibition upregulates PD-L1 and ENT1 expression in cancer cells by regulating HIP1R 4. Pin1 inhibition makes aggressive PDAC eradicable by synergizing with immunotherapy and chemotherapy	[241]
N^6^-methyladen-osine (M^6^A) Demethylase (ALKBH5)	Upregulation	Intrahepatic cholangiocarcinoma (ICC)	ALKBH5 inhibitor + anti-PD-1 therapy	1. PD-L1 is regulated by ALKBH5, which is a direct target of m6A modification 2. ALKBH5 deficiency promotes PD-L1 mRNA degradation 3. ALKBH5 suppresses anti-tumor T cell immunity in PD-L1-dependent manner 4. ALKBH5 and PD-L1 is positively correlated in clinical ICC specimens	[242]
Peripheral serotonin	Upregulation	CRC and pancreatic cancer	Peripheral serotonin inhibitor + anti-PD-1 therapy	1. Platelet-derived serotonin enhances growth of murine MC38 and Panc02 tumors 2. Peripheral serotonin impairs accumulation of CD8^+^ T cells within mouse tumors and dampens the function of CD8^+^ T cells 3. Serotonin promotes tumor growth in mouse models by enhancing PD-L1 expression 4. Pharmacological inhibition of serotonin dampens growth of tumors and enhances efficacy of anti-PD-1 therapy in mice	[223]
EGF/EGFR	EGF/EGFR stimulates PD-L1 glycosylation	TNBC	Drug-conjugated glycosylated-PD-L1(gPD-L1) antibody	1. N-linked glycosylation is required for interaction between PD-L1 and PD-1 2. EGF/EGFR induces PD-L1 glycosylation via B3GNT3 glycosyltransferase 3. gPD-L1 antibody stimulates PD-L1 internalization 4. gPD-L1-ADC shows potent toxicities to cancer cells and bystander effects	[243]
MYC	Upregulation	Leukemia and lymphomas	Not applicable	1. MYC regulates the expression of CD47 and PD-L1 2. Suppression of MYC in tumor cells caused reduced mRNA and protein levels of CD47 and PD-L1 3. MYC directly bind to the promoters of the CD47 and PD-L1	[94]
MUSE for MT-CRISPR/Cas9 (targeting both PD-L1 and CD47)	Downregulation	Melanoma	Not applicable	1. MUSE synergistically boosting CD8^+^ T cells and M1 macrophages-mediated adaptive and innate anticancer immunity 2. The MUSE-nano-CRISPR system showed efficient disruption efficiency of PD-L1 and CD47 in vitro and in vivo	[237]
Multispecific Platinum (IV) Complex DNP	Downregulation	Breast cancer (BC)	Not applicable	1. COX-2 plays an important role in the progression of breast cancer, correlating with the levels of PD-L1 2. DNP reduces the expression of COX-2 and PD-L1 in vitro and in vivo 3. DNP displayed potent antitumor activity and almost no general toxicity in mice bearing TNBC	[244]
Curcumin	Downregulation	HCC	anti-PD-1 therapy	1. Curcumin reduced P300-induced histone acetylation in the promoter region of TGF-β1, thereby inhibiting PD-L1 expression 2. The combination of curcumin and anti-PD-1 antibodies showed better anticancer effects in vitro and in vivo by activating lymphocytes, inhibiting immune evasion and downregulating TGF-β1 expression	[245]
TAM-targeted biomimetic nano-RBC system	Downregulation	TNBC and CRC	Not applicable	1. TAM depletion and hypoxia alleviation with TAM-targeted biomimetic nano-RBC system synergistically reprogram the immunosuppressive TME 2. This system downregulates PD-L1 expression of tumor cells, decreases immunosuppressive cytokines and increases the immunostimulatory IFN-γ and boost CTL response	[246]
Sunitinib	Downregulation	Melanoma and NSCLC	Anti-CTLA-4 therapy	1. Sunitinib modulates the expression of tumor PD-L1 via p62, which binds to PD-L1 and specifically enhances its translocation into autophagic lysosomes for degradation 2. Sunitinib showed synergistic anticancer efficacy with CTLA-4 blockade in immunocompetent mice models of melanoma and NSCLC by increasing tumor-infiltrating T cell activity	[247]

*TNBC* triple negative breast cancer, *CRC* colorectal cancer, *NSCLC *non-small-cell lung cancer, *BC* breast cancer, *HCC* hepatocellular carcinoma, *MV* measles virus, *B3GNT3* b-1,3-N-acetylglucosaminyl transferase, *ADC* antibody–drug conjugate, *MUSE* multistage sensitive nanocomplex, *COX-2* cyclooxygenase-2, *Erk1/2* extracellular signal-regulated kinases ½, *TAM* tumor-associated macrophage, *nano-RBC* nano-red blood cell, *IFN* interferon, *mAb* monoclonal antibody

#### Endogenous factors

Endogenous factors refer to changes in oncogenes or tumor suppressor genes that enhance the expression of PD⁃L1 in cancer cells, such as overexpression of *MYC*, mutation of the RAS oncogene and activation mutation of the EGFR, which can upregulate PD-L1 expression and thus promote immune escape.

The transcription factor Myc is usually overexpressed in human cancers and regulates many genes associated with cell proliferation and survival [186]. Casey et al. [94] found that Myc directly activated the transcription of CD47 (also known as IAP) and PD-L1, which are involved in innate and adaptive immune escape. CD47 is an antiphagocytic protein that is overexpressed in multiple types of cancers and transmits a “do not eat me” signal to macrophages and DCs [188, 190]. The expression levels of CD47 and PD-L1 are related to anti-angiogenesis and the induction of senescence in T cell acute lymphoblastic leukemia (T-ALL) cells [190].

The RAS-EGFR pathway is a classic intracellular signaling pathway, and carcinogenic RAS signaling has been shown to regulate the mRNA stability of PD-L1 to promote tumor immune reactivity [95]. In TNBC, EGF-induced interaction between PD-L1 and PD-1 requires the expression of β-1,3-N-acetylglucosaminyl transferase (B3GNT3). Downregulation of B3GNT3 can enhance the antitumor immune effect of cytotoxic T cells. Monoclonal antibodies against glycosylated PD-L1 (gPD-L1) blocked the PD-L1/PD-1 interaction and promoted the internalization and degradation of PD-L1 [243].

Caspase 8 is a caspase involved in cell apoptosis and other cellular behaviors. Its mutation is linked with increased cancer risk, and low expression of Caspase 8 is closely correlated with poor prognosis. Caspase 8 induces the degradation of PD-L1 by upregulating TNFAIP3 (A20) expression, which is an ubiquitin editing enzyme that results in PD-L1 ubiquitination. Caspase 8 is a promising biomarker for predicting the sensitivity to anti-PD-L1/PD-1 immunotherapy [219].

Multispecific platinum (IV) complex DNP exhibits high cytotoxicities and anti-inflammatory properties that are superior to those of NP (another multispecific platinum [IV] complex), cisplatin and naproxen. Cyclooxygenase-2(COX-2) plays an important role in the progression of breast cancer, correlating with the levels of PD-L1. Mechanistic studies revealed that DNP reduces the expression of COX-2 and PD-L1 in vitro and in vivo, suppresses the secretion of prostaglandin, reduces the expression of BRD4 and phosphorylated Erk1/2 and blocks the oncogene c-Myc in breast cancer cells [244].

The targets of sunitinib and inhibitory immune checkpoints and suppressive immune cells were significantly positively correlated. Sunitinib modulates the expression of tumor PD-L1 via p62, which binds to PD-L1 and specifically enhance its translocation into autophagic lysosomes for degradation. Sunitinib showed synergistic anticancer efficacy with CTLA-4 blockade in immunocompetent mice models of melanoma and NSCLC by increasing tumor-infiltrating T cell activity. In anti-PD-1-treated NSCLC patients, higher PD-L1 levels and lower p62 levels was observed in the tumor of responders compared to those of nonresponders [247].

#### Signaling pathway changes

Multiple oncogenic pathways are involved in the posttranscriptional regulation of PD-L1FGFR2 is highly expressed in CRC and upregulated PD-L1 expression in CRC xenograft in the mice through the JAK/STAT pathway [191]. Loss of function or mutations of the JAK/STAT pathway induce loss of PD-L1 expression in cancer cells, leading to primary and acquired resistance to anti-PD-1 antibodies. In addition, the inactivated IFNGR/JAK/STAT pathway is detected in recurrent patients following ICB [192]. The PTEN/PI3K/AKT/mTOR pathway is responsible for the transcription of PD-L1. PTEN deficiency or mutations of PIK3CA upregulate PD-L1 expression by activating the AKT/mTOR pathway in glioma, breast cancer and prostate cancer [193]. Interestingly, upregulated PD-L1 expression in a mouse model of lung squamous cell carcinoma accelerated PTEN deficiency [194]. Thus, PTEN has been suggested to interact with PD-L1 in cancer.

Ketogenic diet activates AMPK pathway through inducing energy changes, which enhances the immunotherapy efficacy by downregulating PD-L1 expression and upregulating expression of IFN and antigen presentation genes [202]. Metformin is able to activate AMPK, which directly phosphorylates S195 on PD-L1. S195 phosphorylation impairs glycosylation of PD-L1, leading to its accumulation in the endoplasmic reticulum and the degradation of endoplasmic reticulum-associated proteins (ERAD). In breast cancer patients treated with metformin, activated AMPK and downregulated PD-L1 expression were observed in the tumor tissue [203]. Blocking the inhibitory signal of PD-L1 by metformin can enhance the activity of CTLs against cancer cells. Therefore, ketogenic diet or AMPK agonists are recommended for combination treatment with immunotherapy in cancer patients.

Curcumin inhibits the growth and reduces surface PD-L1 expression in Hep3B cells. Curcumin has a synergistic effect with anti-PD-1 antibodies in slowing Hep3B cell proliferation, activating lymphocytes, inhibiting immune evasion and downregulating TGF-β1 expression. Curcumin inhibits thrombin to reduce P300-induced histone acetylation in the promoter region of TGF-β1, which is known to induce PD-L1 expression. Anti-PD-1 antibodies suppress the binding of PD-1 and PD-L1 to promote anticancer immune activity. Therefore, the combination of curcumin and anti-PD-1 antibodies showed better anticancer effects in vitro. The combination also slowed tumor growth and improved the TME in mouse model of HCC [245].

#### External factors

Proinflammatory cytokines in the TME inhibit antitumor immunity. IFN-γ and TNF-α are two key factors for triggering immunosuppression and resistance to immunosurveillance of T cells [85, 179–181].

IFN-γ exerts its critical role in cancer through the JAK/STAT1/interferon regulatory factor 1 (IRF-1) pathway [204]. The IFN-γ pathway is important in inducing PD-L1 expression in the TME. Endogenous IFN-γ has been reported to upregulate PD-L1 expression in head and neck squamous cell carcinoma through the IFNAR1/STAT1 pathway, thereby promoting immune escape [209]. JAK1/JAK2 inhibitor ruxolitinib inhibits the IFN-γ pathway, which enhances anti-PD-1 efficacy by downregulating PD-L1 expression in MDSCs [238].

Both cancer cells and IFN-γ-induced expression of PD-L1 are dependent on the mTOR pathway. The AKT/mTOR pathway promotes immune escape by driving PD-L1 expression [175]. Therefore, combination treatment with mTOR inhibitors and ICIs may enhance the efficacies of immunotherapies.

NF-κB p65-induced COP9 signalosome 5 (CSN5) is essential for maintaining TNF-α-induced stability of PD-L1 in cancer cells. CSN5 inhibits the ubiquitination and degradation of PD-L1. By downregulating CSN5, curcumin enhances the sensitivity of cancer to anti-CTLA-4 treatment and the function of antitumor T cells by downregulating PD-L1 expression, thus alleviating cancer growth [239].

Intra-tumoral copper levels promoted PD-L1 expression at mRNA and protein levels in tumor cells. Copper chelator downregulates PD-L1 expression by inhibiting the response of cancer cells to proinflammatory cytokines such as IFN-γ, TNF-α and TNF-α/β. Copper-chelating drugs inhibits the expression of PD-L1 by downregulating phosphorylated STAT3, EGFR, AKT and GSK3β and mediates the ubiquitination and degradation of PD-L1 in cancer cells [179]. Dietary composition also affects PD-L1 expression. Epigallocatechin gallate (EGCG), the most abundant ingredient in green tea, downregulates PD-L1 expression in NSCLC induced by IFN-γ and EGF [240].

TAM depletion and hypoxia alleviation synergistically reprogram the TME. This combination concurrently downregulates PD-L1 expression in tumor cells, decreases the levels of immunosuppressive cytokines such as IL-10 and TGF-β, elevates immunostimulatory IFN-γ, enhances the CTL response and boosts the memory response. TAM-targeted chemoimmunotherapy markedly inhibit cancer metastasis and recurrence [246].

#### Others

In addition to the abovementioned mechanisms, other mechanisms are involved in the anticancer effects through regulation of PD-L1. Unique proline isomerase Pin1 drives immunosuppressive TME by influencing CAFs and induces lysosomal degradation of PD-L1. Inhibition of the Pin1 simultaneously blocks multiple cancer pathways, disrupts the immunosuppressive TME and upregulates the expression of PD-L1 and gemcitabine transporter ENT1, thus benefiting PDAC patients undergoing immunochemotherapy [241].

N^6^ methyladenosine (m^6^A) is an important posttranscriptional regulator. ALKBH5 is an m^6^A demethylase that coordinates PD-L1 expression in human intrahepatic cholangiocarcinoma (ICC). N^6^-methyladenosine sequencing (m^6^A-seq) confirmed that PD-L1 mRNA is the direct target of m6A modification, which is regulated by ALKBH5. ALKBH5 inhibits T cell expansion and cytotoxicity by stabilizing the expression level of PD-L1 in cancer cells [242].

Serotonin [5-hydroxytryptamine (5-HT)] is an inflammatory mediator associated with the proliferation and invasion of multiple types of cancer cells [248, 249]. Serotonin promoted expression of PD-L1 on cancer cells in vitro via serotonylation and its levels at metastatic sites of abdominal cancer were negatively correlated with the proportion of tumor-infiltrating cytotoxic T cells. Depletion of serotonin cargo enhanced CD8^+^ T cell infiltration and decreased PD-L1 expression. Pharmacological serotonin depletion enhances anticancer effects of PD-1 inhibitors in mice with colorectal and pancreatic cancer [223].

## Preclinical models used in research about PD-1/PD-L1 blockade

The success of PD-1/PD-L1 blockade in cancer treatment is inseparable from the foundation laid by preclinical experiments. In preclinical research, the selection of tumor cells and animal models is critical to obtain clinically translational data. Therefore, we will briefly describe the tumor cells and animal tumor models used in preclinical studies on PD-1/PD-L1 interaction. PD-1/PD-L1 inhibitors have high response rates in melanoma relative to other cancer types. Lung cancer is currently the second most common cancer, and some PD-1/PD-L1 inhibitors have also achieved good therapeutic effects in specific lung cancer patients. Table 4 summarizes the representative cell lines and animal models for PD-1/PD-L1 interaction studies in melanoma and lung cancer. The information in the table indicates that many mouse and human tumor cells were used in in vitro experiments. For studies in mice, most experiments established xenograft models using mouse tumor cells in immunocompetent C57BL/6 mice and BALB/c mice. Other studies used NSG mice with/without human CD34^+^ human stem cell-engrafted to establish xenograft models of human tumor cells [250, 251]. However, one of the limitations of the xenograft model is that it is too far from the real process of tumorigenesis, and the conclusions obtained in those models cannot be better translated into clinical research. Therefore, the regulation of PD-1/PD-L1 signaling pathway has also been investigated using a transgenic mouse tumor model [252]. Several studies have also established metastasis models by i.v. injection to study the therapeutic effect of PD-1/PD-L1 blockade on tumor metastasis [253, 254]. Most studies have examined the effects of modulating PD-1/PD-L1 in tumor therapy, some of which include the combination of PD-1/PD-L1 blockade with other therapeutic strategies [251, 255].

**Table 4:** Representative cell lines and animal models for PD-1/PD-L1 interaction studies in melanoma and lung cancer

Cancer type	Cancer cell line	Mouse strain	Method for establishing mouse tumor model	Immunotherapy strategy or other treatment	References
Colon cancer	CT26, SK-MEL-28, B16-F10	BALB/c mice, NSG mice	Xenograft models	Anti-PD-L1Ab	[270]
Lung cancer	A549, LL/2	C57/BL/6 mice	Intravenous (i.v.) injection	Anti-PD-1Ab	[253]
Lung cancer	HCC827	BALB/c nude mice	Xenograft models	Photothermal therapy	[271]
Lung cancer	LLC,	C57BL/6 J mice	Xenograft models	Anti-PD-1mAb, chemotherapy	[272]
Lung cancer	CMT167, LL/2, LL/2-luc-M38	C57BL/6 mice and green fluorescent protein (GFP)-expressing mice [C57BL/6-Tg(UBC-GFP)30Scha/J]	Orthotopic tumors and subcutaneous tumors	PD-1/PD-L1 antibody	[273]
Lung cancer	NCI-H460, A549	n.a	n.a	PD-L1 regulation with siRNA	[274]
Lung cancer	A549, H1299	n.a	n.a	PD-L1 knockdown with shRNA	[275]
Lung cancer	A549, PC-9	NOD/SCID mice	Patient-derived xenograft mice	PD-L1 knockdown with shRNA	[276]
Lung cancer and other cancer types	PC-3,A549, HepG2, SiHa	NU/J male mice, NSG mice	Xenograft models	CAR T cell, oncolytic adenovirus, anti-PD-L1Ab	[251]
Lung cancer and other cancer types	LLC, EL4, EL4-OVA, MC-38, MC38-OVA	C57BL/6 mice, BALB/c mice	Xenograft models	Anti-PD-L1Ab, anti-CTLA-4Ab	[277]
Lung cancer and other cancer types	NCI-H292, HCC827, OV79, A204	NSG mice, NOG mice	Xenograft models	Anti-PD-L1 monoclonal antibody LY3300054	[278]
Lung cancer and other cancer types	LLC, H1944, CALU-6, H2030, H441, H358, Hop62	C57BL/6J mice, DBA/2 mice	Xenograft models	Anti-PD-1Ab, Targeted therapy	[279]
Lung cancer and other cancer types	PC-9, SW480, MC38	C57BL/6	Xenograft models	Anti-PD-1Ab, anti-PD-L1Ab	[280]
Lung cancer and other cancer types	HT1080, A549, MCF-7, NIH:OVCAR-3, PANC-1	n.a	n.a	Anti-PD-1Ab	[281]
Lung cancer and other cancer types	A375, A549, CAKI-1, H1299, H1975, HCC827, HCT116, KU-19–19, MDA-MB-231, RKO	NSG mice	Xenograft models	Anti-PD-L1Ab	[282]
Lung cancer, breast cancer, liver cancer	A549/CDDP, MCF7/ADR, HepG2/ADR	BALB/C nude mice	Xenograft models	Anti-PD-L1Ab	[283]
Lung cancer, Melanoma and other cancer types	B16-F10, CT26, 4T1, L12, A549, H1299, PC9	C57BL/6 mice, BALB/c mice	Xenograft models, intraperitoneally (i.p.) injection	Other treatment	[284]
Lung cancer, melanoma and other cancer types	MCF-7, MDA-MB-231, HepG2, A549, A375, HeLa	n.a	n.a	Targeted therapy	[285]
Lung cancer, melanoma, colorectal cancer, breast cancer	PC-9, A375, SW116, H1975, 4T1	n.a	n.a	PD-L1 knockdown with shRNA, SiRNA, plasmid and small molecule drug(PD-L1/PD-1 inhibitor 1, BMS1166)	[286]
Lung cancer, melanoma, prostate cancer	HCC827, A549, H2228, PC9, LLC, A375, A431, SK-MEL-5, SK-MEL-28, DU145	C57BL/6	Xenograft models	Anti-PD-L1Ab, PD-L1 knockdown with SiRNA	[287]
Lung cancer, prostate cancer	NCI-H460, A549, PC-3, NCI-H1299, NCI-H446	n.a	n.a	PD-L1 detection	[288]
Lung cancer, TNBC	A549, MDA-MB-231	NSG mice	Xenograft models	Anti-CD137 × PD-L1 bispecific Ab, anti-PD-1Ab, anti-PD-L1Ab	[250]
Melanoma	A375, A431	n.a	n.a	Other treatment	[289]
Melanoma	B16-F0, B16-F1	n.a	n.a	Anti-PD-L1Ab	[290]
Melanoma	B16-PD-L1	C57BL/6 mice	Xenograft models	Chemotherapy, other treatment	[255]
Melanoma	PD-L1 KO B16-OVA	C57BL/6J mice, hPD-L1 KI mice	Xenograft models	Anti-hPD-L1Ab, anti-CD8Ab, anti-CD4Ab, anti-F4/80Ab	[291]
Melanoma	B16-F10	C57BL/6 mice	Xenograft models	Anti-PD-L1Ab	[292]
Melanoma and other cancer types	HepG2-Luc, HepG2, B16	C57BL/6 mice	Xenograft models	Other treatment	[293]
Melanoma and other cancer types	AT-3, 4T1, B16-F10, MC38,	C57BL/6 mice, Pmel-1 TCR-transgenic mice, Batf3 − / − mice, BALB/c − AnNCr mice	Xenograft models, orthotopic model	Anti-PD-L1Ab,anti-CD4Ab, anti-CD8βAb, anti-NK1.1Ab, anti-IL-12p40Ab, anti-IFN-γAb, Targeted therapy, Radiotherapy	[294]
Melanoma, lung cancer and other cancer types	B16, LLC, GL261, MC38, Pan02, MethA, CT26	C57BL/6 N and BALB/c inbred mice	Bilateral xenograft models model	Anti-PD-L1Ab	[295]
Melanoma, Cervical cancer, Colorectal cancer	HeLa, HCC827, PC3, NCI-H2023, 4T1, B16.F10, CT26	BALB/c	Xenograft models	Anti-PD-1Ab, anti-PD-L1Ab, PD1-Fc-OX40L	[296]
Melanoma, Colon cancer	MC38-hPD-L1, A375	NOG mice, human PD-L1/LAG-3 double knock-in mice	Xenograft models	Anti-PD-L1 × LAG-3 bispecific Ab, anti-PD-L1Ab	[252]
Melanoma, Colon cancer	B16, CT26	C57BL/6 mice, BALB/c mice	Genetically modified mouse model, xenograft models, intravenous (i.v.) injection	Anti-PD-1Ab	[297]
Melanoma, Colon cancer	B16-F10, CT26	C57BL/6 mice, BALB/c mice	Xenograft models	Anti-PD-L1 mAb, anti-TRP-1 mAb	[298]
NSCLC	HCC827, H1975, PC-9	BALB/c athymic nude mice	Xenograft models, i.v. injection	PD-L1 knockdown with shRNA	[254]
NSCLC	HCC827, H1975	n.a	n.a	Targeted therapy	[299]
NSCLC	A549, H460, H1975	C57/BL/6 mice	Xenograft models	PD-L1 knockout	[300]
NSCLC	A549, NCI-H23, NCI-H460, NCI-H226	n.a	n.a	PD-L1 quantification on tumor biopsies	[301]
PD-L1-positive solid tumors	K562, Jurkat, A549, NCI-H292, SKOV3, HEK293T	B-NDG mice	Xenograft dual-tumor models	CAR T cell, PD-L1 chimeric costimulatory receptor	[302]

*n.a.* not applicable, *NSCLC* non-small-cell lung cancer, *Ab* antibody

Organoids are tiny, self-organized three dimensional multicellular in vitro tissue construct that displays realistic micro-anatomy and mimics their corresponding in vivo organs. Such cultures have the ability to replicate much of the complexity of an organ and recapitulate certain functions of the represented organ [256]. Reliable methods for predicting treatment response are urgently needed in clinical oncology. Cancer organoids can accurately reproduce important genetic and phenotypic characteristics of the tissue from which they are derived, tumor subtypes, and maintain intra- and inter-tumor heterogeneity, and thus have the potential to be used to predict individualized treatment response [257]. In recent years, many studies have used cancer organoids, especially patient-derived cancer organoids (PDO) to conduct comprehensive studies of PD-1/PD-L1 interaction. Table 5 summarizes this aspect of research conducted in cancer organoids. Most cancer organoids were derived from cancer tissues of patients with gastrointestinal tumors. Organoid/immune cell co-cultures can model tumor-immune microenvironment. Most studies used cancer organoids to mimic the interaction of cancer cells with the human immune system in vitro. When conducting in vivo studies, NSG mice are required. After establishing tumor models in mice by orthotopic transplantation, these cancer organoids can predict the efficacy of PD-1/PD-L1 blockade as well as other treatment regimens [258–261]. These studies used organoids to investigate PD-1/PD-L1 interaction in various aspects, including testing the anticancer efficacy of PD-1/PD-L1 inhibitors [261–263], analyzing the regulation mechanism of PD-L1 expression [258, 259], finding strategies to enhance the therapeutic effect of PD-1/PD-L1 blockade [260, 264–268], finding new immune checkpoints [269]. Through these studies, we can see that cancer organoids can be used to simulate the immune microenvironment in cancer patients, providing an effective tool for improving the efficacy of PD-1/PD-L1 blockade.

**Table 5:** Published studies that investigated PD-L1/PD-1 signal pathways in cancer organoids

Cancer type	Source of cancer organoids	Mouse strain receiving organoid	Method for establishing mouse tumor model from organoid	Immunotherapy strategy or other treatment in cancer organoid	References
CRC	Biopsies from patient	n.a	n.a	Anti-PD-L1 Ab	[264]
CRC	Tumor tissue from patient	n.a	n.a	Pembrolizumab, nivolumab, atezolizumab	[262]
CRC	Tumor tissues of dMMR CRCs	n.a	n.a	Anti-PD-1 Ab, Anti-DKK1 Ab	[265]
Diverse tumor types	Biopsies from patient or mouse tumors (B16-SIY, MC38, A20-OVA)	NSG mice	Injected subcutaneously	Anti-PD-1 Ab, anti-PD-L1 Ab	[261]
Gastric cancer	Biopsied or resected tumor tissues	NSG mice	Orthotopic transplantation	Nivolumab, targeted therapy	[258]
Gastric cancer	Tumor tissue from patient	NSG mice	Orthotopic transplantation	Nivolumab, targeted therapy	[259]
Gastric cancer	human gastric tissue, induced pluripotent stem cells	n.a	n.a	Targeted therapy, nivolumab	[267]
Gastric cancer	Gastric glands from normal mouse stomach, cancer tissue of transgenic mouse	n.a	n.a	n.a	[303]
Gastric cancer	Tumor tissue from patient	n.a	n.a	Dexamethasone, pembrolizumab,	[268]
NSCLC	Biopsies from patient	n.a	n.a	Anti-PD-L1 Ab (atezolizumab, avelumab), targeted therapy	[266]
Ovarian cancer	Tumor tissue from patient	n.a	n.a	Bispecific anti-PD-1/PD-L1 antibody, pembrolizumab, anti-PD-L1 Ab (LY3300054)	[263]
Pancreatic cancer, TNBC	Tumor tissue from patient and mouse	n.a	n.a	Anti-PD-1, PD-L1 and TIM3 Ab or NKG2A, TIM3, TIGIT and LAG3 protein	[269]
PDAC	Tumor tissue from patient and mouse	NSG mice	Orthotopic transplantation	Anti-PD-1 Ab, targeted therapy, chemotherapy	[260]

*n.a.* not applicable, *CRC* colorectal cancer, *NSCLC* non-small-cell lung cancer, *TNBC* triple negative breast cancer, *PDAC* pancreatic ductal adenocarcinoma, *dMMR* different mismatch repair, *NSG* NOD scid gamma, *Ab* antibody

## Adverse events of ICIs

Although ICIs induce the immune system to fight against cancer cells by activating T cells, they may also help to attack normal cells and thus result in immune-related adverse events (irAEs). The infiltration of immune cells, especially T cells, caused by combination treatment with anti-CTLA-4 and anti-PD-1/PD-L1 antibodies leads to irAEs.

Some irAEs caused by immunotherapy may be similar to AEs of other therapeutic strategies. However, similar AEs (e.g., diarrhea, enteritis, rashes and itching) can be caused by different mechanisms. The occurrence of irAEs is related to inflammatory responses, especially those mediated by CD8^+^ T cell activation. Other types of inflammatory cells such as Th17 may also be involved. An immunohistochemistry assay revealed the infiltration of CD4^+^ and CD8^+^ T cells in damaged skins and organs, and highly activated effector T cells are correlated with the incidence of AEs [29, 304–306]. Generally, irAEs are classified into organ-specific AEs (e.g., colitis, hepatitis and pneumonia), common AEs (e.g., fatigue, diarrhea and rashes) and others related to systemic inflammation. Most irAEs are mild to moderate, but serious or life-threatening irAEs have also occurred, with the highest fatality rates due to AEs in the nervous system and heart [307].

Compared with AEs caused by conventional chemotherapy, irAEs are characterized by delayed onset, long-term duration and different toxicity spectra. Pneumonia and arthralgia are the most common irAEs [29, 307–310]. The incidence of all-grade AEs caused by PD-1/PD-L1 inhibitors is lower than that of chemotherapy, and that of grade 3–4 AEs accounts for 7–13% with a relatively high safety [310–312]. Although the incidence of AEs increases in combination treatment, most of them are well tolerated [130, 313]. At present, management strategies for irAEs have been published, and most irAEs can be controlled or even reversed by withdrawal with or without corticosteroid hormone medication [309, 314].

Compared with those of conventional treatment, AEs caused by ICIs mainly affect the skin, endocrine system and lungs [315, 316]. Of the common ICIs, nivolumab is considered the safest, followed by atezolizumab, pembrolizumab, ipilimumab and tremelimumab. Their main AEs are summarized as follows: atezolizumab (hyperthyroidism, nausea and vomiting), nivolumab (endocrine toxicity), pembrolizumab (arthralgia, pneumonia and hepatotoxicity), ipilimumab (skin, gastrointestinal and kidney toxicity) and tremelimumab (rashes, diarrhea and fatigue). Taken together, these findings indicate that nivolumab is the safest ICI that is specifically suitable for lung cancer treatment. In conclusion, irAEs are insidious onset, lack specificity and have a wide spectrum of toxicity. Clinicians need to strengthen the management of irAEs from five aspects: prevention, assessment, examination, treatment and detection, so as to effectively control the disease.

## Conclusion

Based on the regulatory mechanisms in T cells, PD-1/PD-L1 blockade has greatly advanced cancer treatment by enhancing the antitumor immune response. Its efficacy in the treatment of melanoma and NSCLC, in particular, is extraordinary, as it achieves long-term remission in a portion of cancer patients without recurrence.

Hot tumors identified by relevant biomarkers, such as T cell infiltration and PD-L1 expression, are closely linked with the clinical benefits of anti-PD-1/PD-L1 antibodies. In cold tumors, anti-CTLA-4 treatment creates a TME that is favorable to anti-PD-1/PD-L1 antibody treatment by recruiting T cells to target cancer lesions and inducing PD-L1 expression, which provides a rationale for combination therapy. Currently, great effort is directed to identifying predictive biomarkers for ICB.

PD-1/PD-L1 blockade has low response rates in many cancer patients due to innate and acquired resistance. Therefore, based on the resistance mechanism, PD-1/PD-L1 blockade combined with other treatment regimens is an effective strategy to improve anticancer efficacy and reduce side effects. Combining approved PD-1/PD-L1 inhibitors with other approved treatments may facilitate rapid approval of an effective combination. On the other hand, combining approved/investigational PD-1/PD-L1 inhibitors with other investigational treatment will lead to many breakthroughs. The expression of PD-L1 in TME also affects the effect of PD-1/PD-L1 blockade. This review introduces the factors affecting PD-L1 expression and strategies to regulate its expression. The success of PD-1/PD-L1 inhibitors in cancer therapy relies on extensive preclinical research. The selection of cell lines, animal strains and cancer models is critical for obtaining translational data. Therefore, this review describes the models used in preclinical studies of PD-1/PD-L1 interaction in melanoma and lung cancer. Notably, many studies have utilized cancer organoids to mimic the interaction of cancer cells with the human immune system in vitro, and these organoids are able to accurately replicate key genetic and phenotypic features of patient cancer tissue while maintaining heterogeneity. They can be used to simulate the immune microenvironment of cancer patients and provide an effective tool for improving PD-1/PD-L1 blockade.

PD-1/PD-L1 blockade and its combination therapy can control or even cure malignant diseases in the long term, providing new insights into cancer treatment. Specific agents or interventions can modulate the level of PD-1 and PD-L1, so as to exert a similar effect to ICIs. Owing to the inherent specificity, adaptability and memory of the immune system, researchers are able to continuously target and precisely kill cancer cells. The next goal of preclinical and clinical research is to find reasonable combinations of PD-1/PD-L1 blockade and other treatments to reduce toxic side effects, exert stronger anti-tumor immune responses and precisely kill cancer cells, so that cancer can become a type of curable chronic disease.

## Data Availability

Not applicable.

## Abbreviations

PD-1: Programmed cell death 1 receptor
PD-L1: Programmed cell death ligand 1
CTLA-4: Cytotoxic T-lymphocyte-associated protein 4
ICB: Immune checkpoint blockade
TME: Tumor microenvironment
ICIs: Immune checkpoint inhibitors
DCs: Dendritic cells
IgV: Ig variable-type
ITIM: Immunoreceptor tyrosine-based inhibitory motifs
ITSM: Immunoreceptor tyrosine-based switch motifs
TCR: T cell receptor
MHCs: Major histocompatibility complexes
APC: Antigen presenting cell
BCR: B cell receptor
NSCLC: Non-small-cell lung cancer
RCC: Renal cell carcinoma
Teff: Effector T cells
Tm: Memory T cells
Treg: Regulatory T cells
Tex: Exhaustion T cells
ECM: Extracellular matrix
EMT: Epithelial-to-mesenchymal transition
LCMV: Lymphocytic choriomeningitis virus
TGF-β: Transforming growth factor β
IFN-α: Interferon-α
ISRE: Interferon-sensitive responsive element
CTLs: Cytotoxic lymphocytes
IFN-γ: Interferon-γ
TNF-α: Tumor necrosis factor-α
ILs: Interleukins
EGFR: Epithelial growth factor receptor
EGF: Epithelial growth factor
T-ALL: T cell acute lymphoblastic leukemia
FDA: Food and Drug Administration
AEs: Adverse events
ORR: Overall response rate
mPFS: Median progression-free survival
irAEs: Immune-related AEs
ICD: Immunogenic cell death
DAMPs: Damage-associated molecular patterns
MDSCs: Myeloid-derived suppressor cells
SC: Systemic chemotherapy
LC: Local chemotherapy
SCLC: Small-cell lung cancer
TNBC: Triple negative breast cancer
IFNs: Type I interferons
SBRT: Stereotactic body radiation therapy
OS: Overall survival
BA: Bintrafusp alfa
BART: Breathing adapted radiotherapy
MPR: Main pathological response
pCR: Pathological complete remission
PR: Partial remission
TKIs: Tyrosine kinase inhibitors
TILs: Tumor infiltrating lymphocytes
DDR: DNA damage response
PARP: Poly ADP-ribose polymerase
CHK1: Checkpoint kinase 1
CRPC: Castration-resistant prostate cancer
MAPKi: MAPK inhibitors
MBM: Melanoma brain metastasis
CSF-1R: Colony-stimulating factor 1 receptor
CRC: Colorectal cancer
TAMs: Tumor-associated macrophages
TIFP: Tumor interstitial fluid pressure
ICT: Immune checkpoint therapy
STING: Stimulator of interferon genes
IFN-I: Type I interferon
ACT: Adoptive cell therapy
MUSE: Multistage sensitive nanocomplex
B3GNT3: β-1,3-N-acetylglucosaminyl transferase
gPD-L1: Glycosylated PD-L1
COX-2: Cyclooxygenase-2
Erk1/2: Extracellular signal-regulated kinases 1/2
Erk1/2: Extracellular signal-regulated kinases 1/2
ERAD: Endoplasmic reticulum-associated proteins
IRF-1: Interferon regulatory factor 1
CSN5: COP9 signalosome 5
EGCG: Epigallocatechin gallate
PDAC: Pancreatic ductal adenocarcinoma
m^6^A: N^6^ methyladenosine
ICC: Intrahepatic cholangiocarcinoma
5-HT: 5-Hydroxytryptamine
TPH: Tryptophan hydroxylase
